# Supplementary material for: Stratification of hypertension and SARS-CoV-2 infection by quantitative NMR spectroscopy of human blood serum
Source: Commun Med (Lond). 2023 Oct 16;3:145. doi: 10.1038/s43856-023-00365-y (PMC11081957; doi:10.1038/s43856-023-00365-y)
Supplement: Supplementary file 2 — Supplementary Information [file 43856_2023_365_MOESM2_ESM.docx]

**Supplementary Information**

**Stratification of hypertension and SARS-CoV-2 infection by quantitative NMR spectroscopy of human blood serum**

Jasmin Kazenwadel^1^, Georgy Berezhnoy^1^, Claire Cannet^2^, Hartmut Schäfer^2^, Tobias Geisler^3^, Anne-Katrin Rohlfing^3^, Meinrad Gawaz^3^, Uta Merle^4^†, Christoph Trautwein^1^*†

^1^ Werner Siemens Imaging Center, Department for Preclinical Imaging and Radiopharmacy, Eberhard Karls University Tuebingen, Germany

^2^ Bruker BioSpin GmbH, Applied Industrial and Clinical Division, Ettlingen, Germany

^3^ Department of Internal Medicine III, Cardiology and Angiology, University Hospital Tübingen, Tübingen, Germany

^4^ Department of Internal Medicine IV, University Hospital Heidelberg, Heidelberg, Germany

***** Author to whom correspondence should be addressed: Christoph.Trautwein@med.uni-tuebingen.de

† These authors contributed equally to this work.

**Supplementary Table 1**

***General abbreviations***

| **Name** | **Extended name** |
| --- | --- |
| ACEI | Angiotensin- converting- enzyme inhibitor |
| AHT | Arterial hypertension |
| AT1RB | Angiotensin-1 receptor blockers |
| AUC | Area under the curve |
| BB | Beta blockers |
| BCAA | Branched chained amino acids (leucine, isoleucine, valine) |
| BP | Blood pressure |
| CCB | Calcium channel blockers |
| FC | Fold change |
| FDR | False Discovery rate |
| HC | Healthy cohort |
| NMR | Nuclear magnetic resonance |
| T2DM | Diabetes mellitus Type 2 |
| PACS | Post-acute-covid- syndrom |
| PCA | Principal component analysis |
| OPLS-DA | Orthogonal partial least squares discriminant analysis |
| RAASI | Renin-angiotensin-aldosterone- system inhibitors |

**Supplementary Table 2**

***Abbreviations of routine laboratory values and NMR values with units***

| **Name** | **Extended name** | **Unit** |
| --- | --- | --- |
| BUN | Blood urea nitrogen | mg/dL |
| Creatinine.lab | Creatinine (laboratory report) | mg/dL |
| CRP | C- reactive protein | mg/L |
| GFR | Glomerular filtration rate | ml/min |
| GGT | Gamma-glutamyl transferase | Unit/L |
| Glyc | Glycoproteins | Procedure defined Unit |
| GOT | Glutamate- oxalacetate- transferase | Unit/L |
| Leuk | Leucozytes | x10^9^cells/L |
| LDH | Lactat dehydrogenase | Unit/L |
| SPC | Supramolecular phospholipid composite | Procedure defined Unit |
| TMAO | Trimethylamine-N-oxide | mmol/L |

**Supplementary Table 3**

**General characteristics of the COVID-19 cohort (Coronataxi cohort)**

This table gives an overview over the COVID-19 cohort composition, which included 329 study participants. The characteristics are separated by gender. BMI body mass index, IQR interquartile range, no number.

| **Characteristics** | **Total** | **Female** | **Male** |
| --- | --- | --- | --- |
| Gender, no. (%) | 329 | 174 (52,9) | 155 (47,1) |
| Age, median (IQR) | 54 (44- 64) | 52 (41- 63) | 58 (48- 65) |
| BMI, median (IQR) | 27.9 (24.4- 32.2) | 26.7 (22.6- 32) | 29 (25.9- 32.3) |

**Supplementary Table 4**

**Disease severity criteria of the Coronataxi cohort**

Hospitalization rate with duration of stay, need for oxygen and intensive care treatment are given in this table, as well as the deadlines during the Coronataxi study. The criteria are separated by gender, respectively. ICU intensive care unit, IQR interquartile range, no number.

| **Disease severity** | **Total** | **Female** | **Male** |
| --- | --- | --- | --- |
| Hospital total, no. (%) | 71 (22.8) | 25 (35.2) | 46 (64.8) |
| Duration of stay, median (IQR) | 6 (3-8) | 4 (3-7) | 6 (4-8) |
| Oxygen demand,  no. (%) | 48 (67.6) | 15 (31.2) | 33 (68.8) |
| ICU, no. (%) | 8 (11.3) | 3 (37.5) | 5 (62.5) |
| Death, no. (%) | 5 (1.5) | 3 (60.0) | 2 (40.0) |

**Supplementary Table 5**

**Pre-existing disorders in the Coronataxi cohort**

The pre- existing diseases were captured through a questionnaire and/or anamnesis. The 17 recorded pre-existing disorders are listed in this table in total numbers and percentages and separately by gender. Additionally, pregnancy and the COVID-19 vaccination were recorded. AF arterial fibrillation, AHT arterial hypertension, CAD: coronary artery disease, COPD: chronic obstructive pulmonary disease, DM diabetes mellitus, OSAS obstructive sleep apnoea syndrome.

| **Pre-existing disorders** | **Total (%)** | **Female (%)** | **Male (%)** |
| --- | --- | --- | --- |
| AHT | 134 (40.7) | 63 (47.0) | 71 (53.0) |
| CAD | 33 (10.0) | 9 (27.3) | 24 (72.7) |
| Chronic heart failure | 6 (1.8) | 4 (66.7) | 2 (33.3) |
| D.M. | 44 (13.4) | 20 (45.5) | 24 (54.5) |
| Asthma | 44 (13.4) | 31 (70.5) | 13 (29.5) |
| COPD | 12 (3.6) | 4 (33.3) | 8 (66.7) |
| OSAS | 22 (6.7) | 7 (31.8) | 15 (68.2) |
| Depression | 23 (7.0) | 15 (65.2) | 8 (34.8) |
| Cancer | 15 (4.6) | 7 (46.7) | 8 (53.3) |
| Cancer in past | 23 (7.0) | 14 (60.9) | 9 (39.1) |
| Stroke in the past | 16 (4.9) | 8 (50.0) | 8 (50.0) |
| Rheumatoid Arthritis | 10 (3.0) | 9 (90.0) | 1 (10.0) |
| Hypothyroidism | 46 (14.0) | 35 (76.1) | 11 (23.9) |
| Inflammatory bowel disease | 6 (1.8) | 5 (83.3) | 1 (16.7) |
| Thrombosis in the past | 27 (8.2) | 16 (59.3) | 11 (40.7) |
| Organ transplantation in the past | 3 (0.9) | 0 (0) | 3 (100) |
| AF | 12 (3.6) | 6 (50.0) | 6 (50.0) |
| Vaccination (BionTech, AstraZeneca, Moderna) | 6 (1.8) | 4 (66.7) | 2 (33.3) |
| Pregnancy | 4 (1.2) | 4 (100) | 0 (0) |

**Supplementary Table 6**

**Overview of premedication in the Coronataxi cohort**

The medication in the COVID-19 cohort was captured by a questionnaire and/or anamnesis. 25 drugs were recorded in total numbers and percentages, thereof mainly antihypertensives in female and male patients. ACE angiotensin- converting enzyme, ASS acetylsalicylic acid, AT1-R angiotensin-1 receptor, DOAC direct oral anticoagulants.

| **Medication** | **Total (%)** | **Female (%)** | **Male (%)** |
| --- | --- | --- | --- |
| Beta-blockers | 61 (18.5) | 31 (50.8) | 30 (49.2) |
| Calcium channel blockers | 32 (9.7) | 14 (43.8) | 18 (56.3) |
| ACE- Inhibitors | 43 (13.1) | 17 (39.5) | 26 (60.5) |
| AT1-R-blockers | 62 (18.8) | 34 (54.8) | 28 (45.2) |
| L-thyroxin | 41 (12.5) | 31 (75.6) | 10 (24.4) |
| ASS | 46 (14.0) | 18 (31.9) | 28 (60.9) |
| Clopidogrel | 5 (1.5) | 0 (0) | 5 (100) |
| Citalopram/Sertraline | 13 (4.0) | 7 (53.8) | 6 (46.2) |
| Mirtazapine | 3 (0.9) | 2 (66.7) | 1 (33.3) |
| Venlafaxine | 2 (0.6) | 2 (100) | 0 (0) |
| Opipramol/ Amitriptyline | 5 (1.5) | 4 (80.0) | 1 (20.0) |
| Pantoprazole | 40 (12.2) | 21 (52.5) | 19 (47.5) |
| Statine | 46 (14.0) | 13 (28.3) | 33 (71.7) |
| Metformin | 20 (6.1) | 7 (35.0) | 13 (65.0) |
| Allopurinol | 14 (4.3) | 1 (7.1) | 13 (92.9) |
| Insulin | 9 (2.7) | 3 (33.3) | 6 (66.7) |
| Oral antidiabetics | 10 (3.0) | 1 (10.0) | 9 (90.0) |
| NOAC | 17 (5.2) | 5 (29.4) | 12 (70.6) |
| Cortisone | 7 (2.1) | 5 (71.4) | 2 (28.6) |
| Adalimumab | 2 (0.6) | 0 (0) | 2 (100) |
| Azathioprine | 1 (0.3) | 1 (100) | 0 (0) |
| Tacrolimus | 3 (0.9) | 3 (100) | 0 (0) |
| Rituximab | 1 (0.3) | 0 (0) | 1 (100) |
| Methotrexate | 5 (1.5) | 4 (80.0) | 1 (20.0) |
| Spray | 29 (8.8) | 16 (55.2) | 13 (44.8) |

**Supplementary Figure 1**


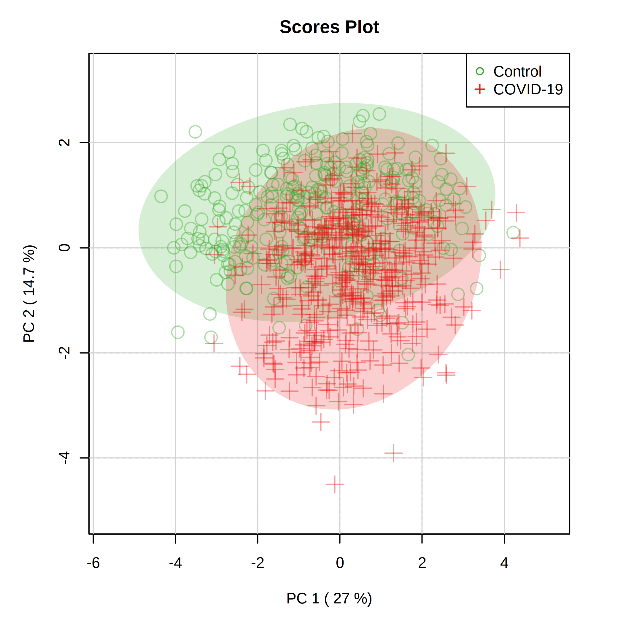

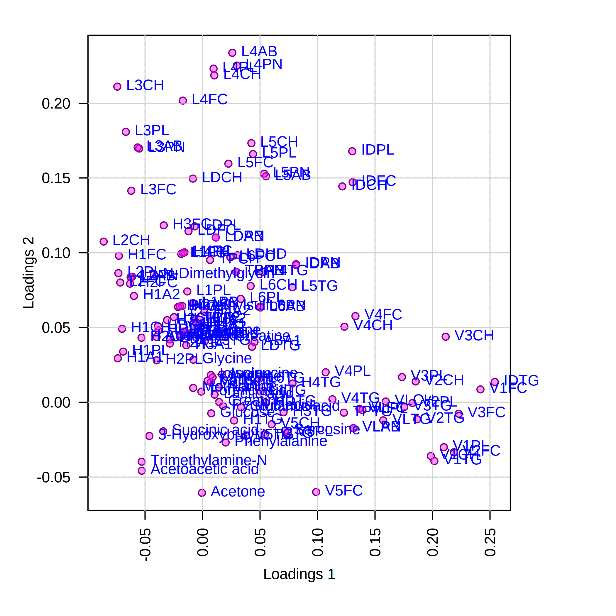

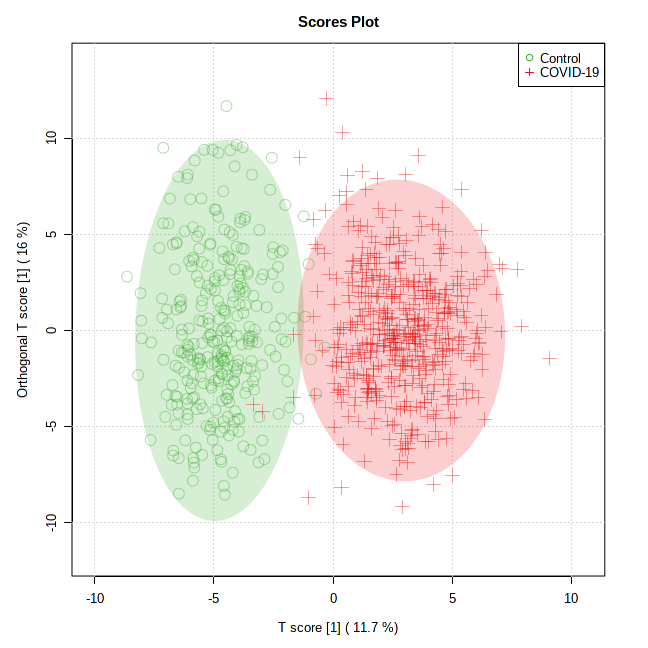

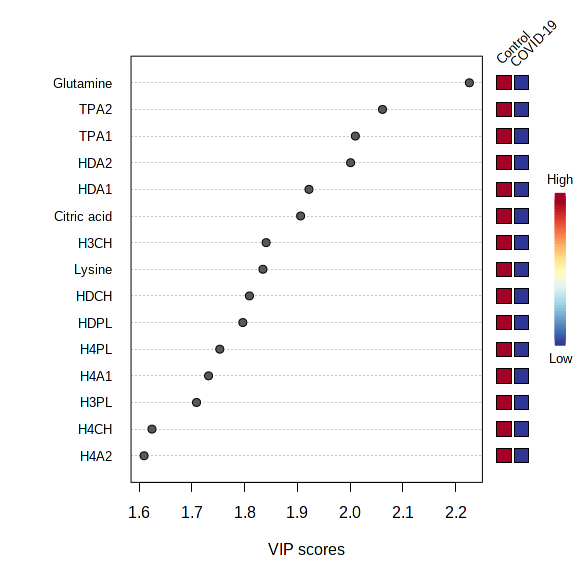


a

b

c

d

**Supplementary Figure 1: Multivariate analysis of the COVID-19 cohort and the control cohort**

The PCA **(a)** gives an overview of the distribution of the COVID-19 cohort (n= 509) with red crosses, and the control cohort (n= 305) with green circles. **(b)** shows the associated loadings plot. The OPLSDA **(c)** illustrates the separation between the individuals from the control cohort and the COVID-19 patients by orthogonal T scores and T scores. The VIP scores plot **(d)** shows the 15 most important lipoproteins which drive the separation of the two cohorts. The legend on the right side of the plot in **(d)** specifies if the lipoproteins are high or low in the respective cohort. OPLSDA orthogonal partial least squares- discriminant analysis, PCA principal component analysis, VIP variable importance in projection.

**Supplementary Figure 2**


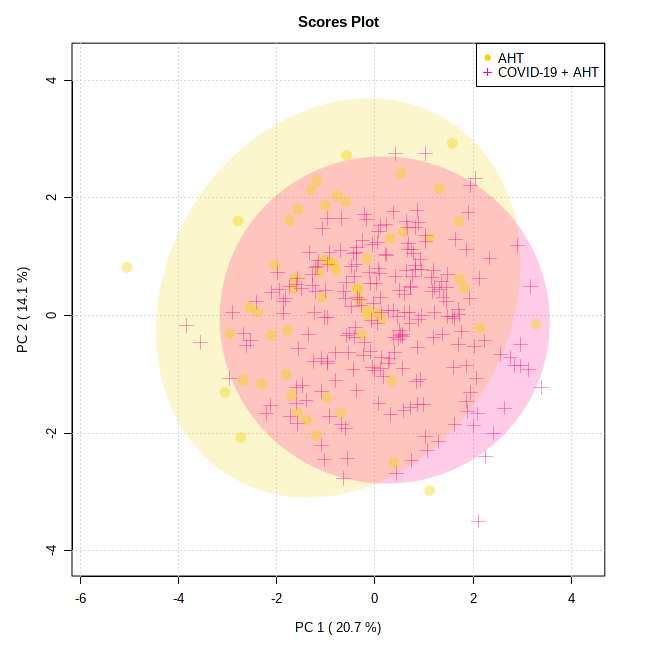

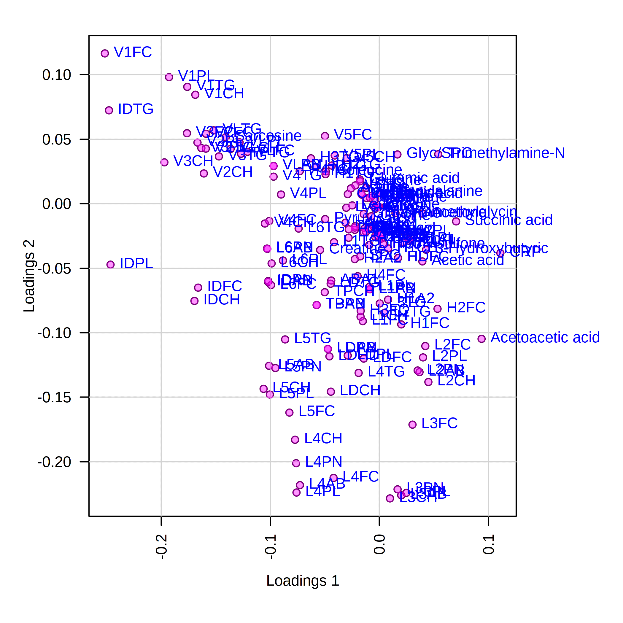

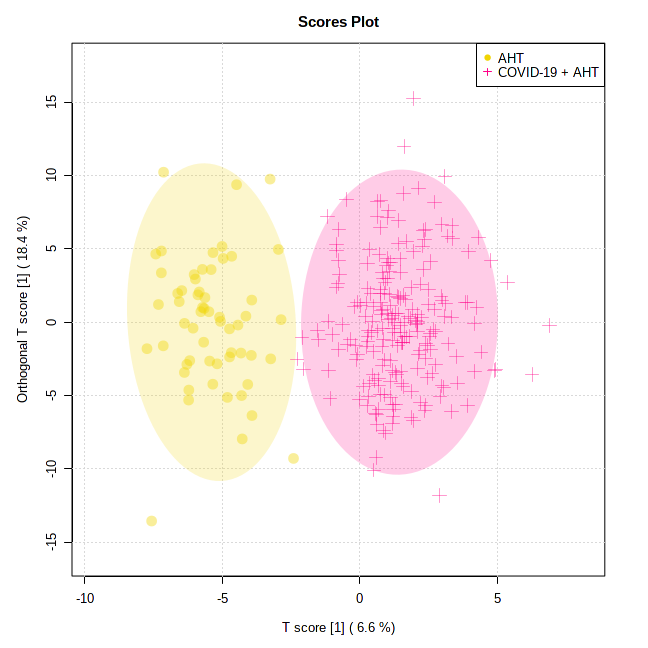

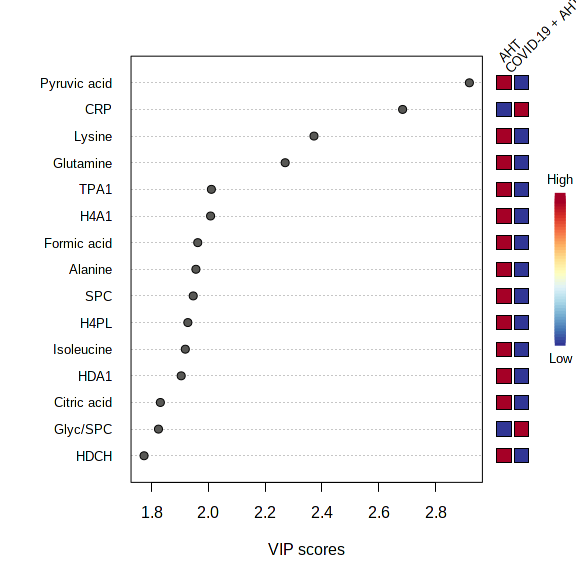


a

b~~a~~

c~~a~~

d~~a~~

***Supplementary Figure 2: Multivariate analysis of the AHT versus the COVID-19 + AHT cohort***

The PCA **(a)** gives an overview of the distribution of the COVID-19 + AHT cohort (n= 216) with pink crosses, and the AHT control cohort (n= 58) with yellow dots. **(b)** shows the associated loadings plot. The OPLSDA **(c)** illustrates the separation between the individuals from the AHT control cohort and the COVID-19 + AHT patients by orthogonal T scores and T scores. The VIP scores plot **(d)** shows the 15 most important lipoproteins which drive the separation of the two groups. The legend on the right side of the plot in **(d)** specifies if the lipoproteins are high or low in the respective cohort. AHT arterial hypertension, CRP C-reactive protein, Glyc glycoproteins, OPLSDA orthogonal partial least squares- discriminant analysis, PCA principal component analysis, SPC supramolecular phospholipid composite, VIP variable importance in projection.

**Supplementary Figure 3**


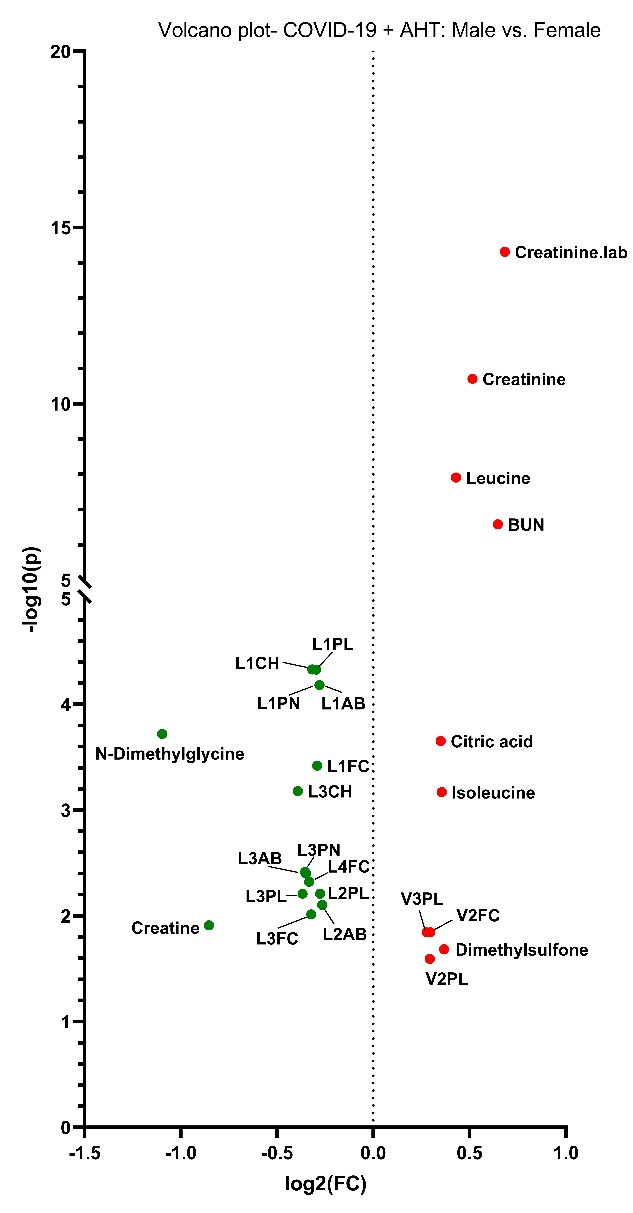

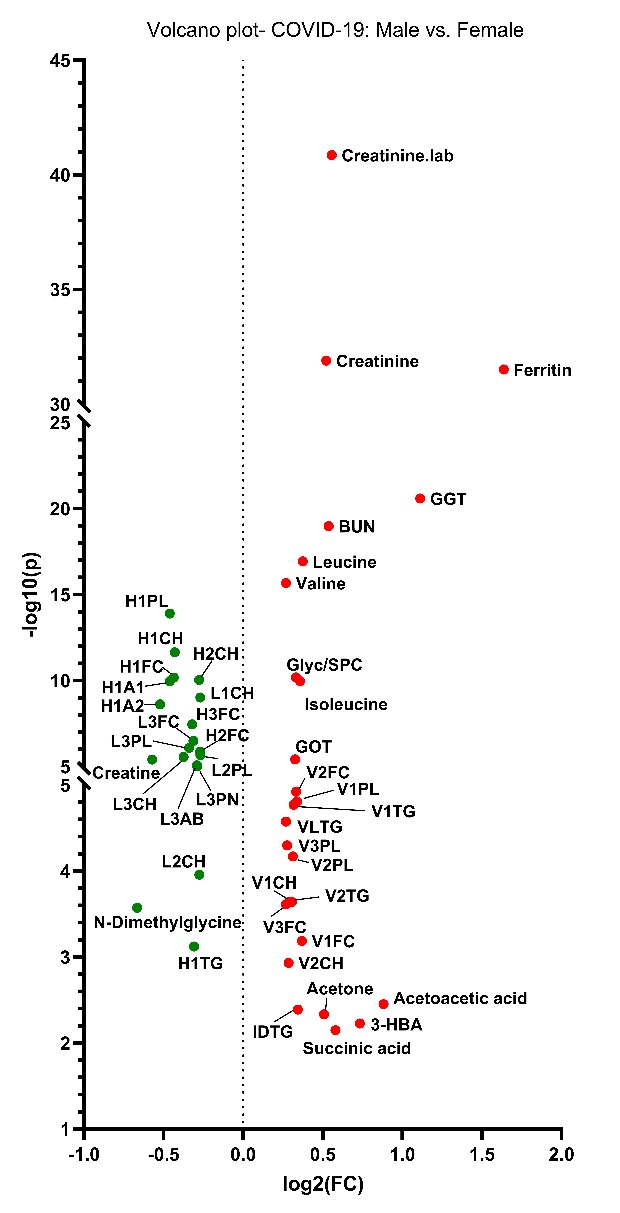

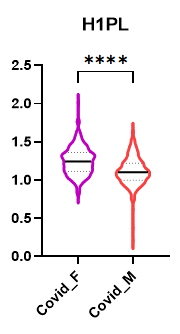


c


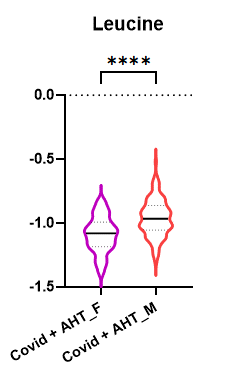

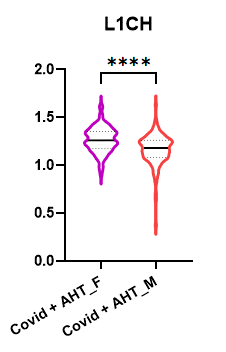


d A volcano plot was also performed for this and 124 samples from 71 men were compared with 92 samples from 63 women.


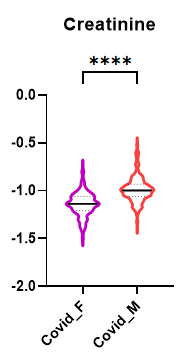


a **A volcano plot was also performed for this and 124 samples from 71 men were compared with 92 samples from 63 women.**

b


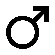

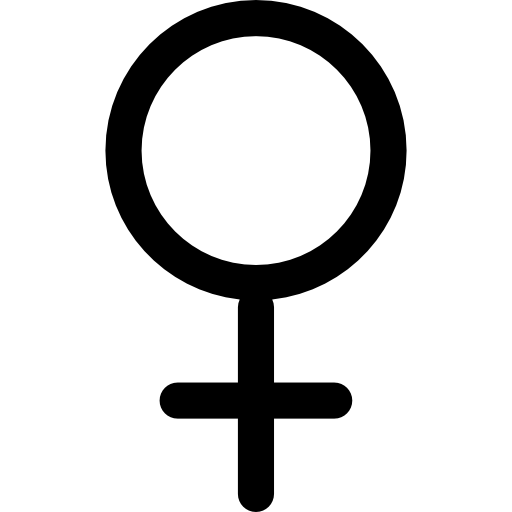

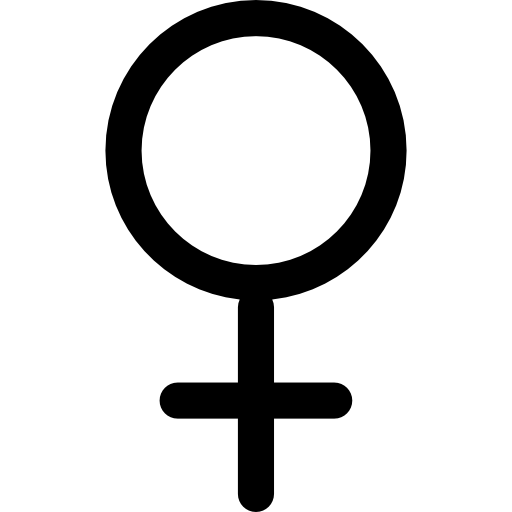

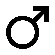

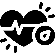

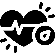

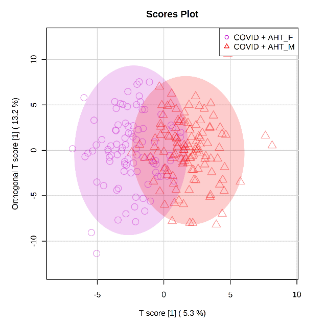

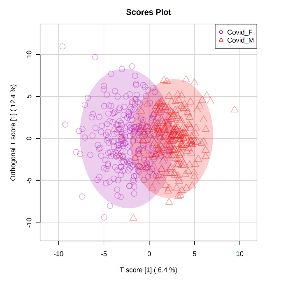


**Supplementary Figure 3: Univariate comparisons by means of volcano plots showing gender differences of COVID-19 patients with and without AHT**

FC > 1.2, p < 0.05, FDR < 0.01: The red features are significantly increased in men, respectively, while the green ones are significantly decreased in men, compared to women. The plots with the orthogonal partial least squares discriminant analysis (OPLS-DA) additionally show to what extent the cohorts can be distinguished from each other. **(a)** shows the comparison between the genders in the COVID- 19 cohort in total (female: n= 255, male: n= 254). **(b)** shows the comparison between the genders in the COVID-19 + hypertension cohort (female: n= 92, male: n= 124). In **(c)** violin plots with max. to min., whisker, and median, show the decreased (framed in green) respectively increased (framed in red) NMR parameters with the highest significance. In **(d)** the left violin plot with max. to min., whisker, and median, shows L1CH, as the lipoprotein with the highest significance, which is decreased in males. The right violin plot shows leucine, as increased NMR parameter in males, with the second highest significance after creatinine. The y-axis shows the normalized concentration. 3-HBA 3-hydroxybutyric acid, AHT arterial hypertension, BUN blood urea nitrogen, Creatinine.lab creatinine from laboratory report, GGT gamma-glutamyl- transferase, GOT glutamate- oxalacetate- transferase, F female, M male.

**Supplementary Figure 4**


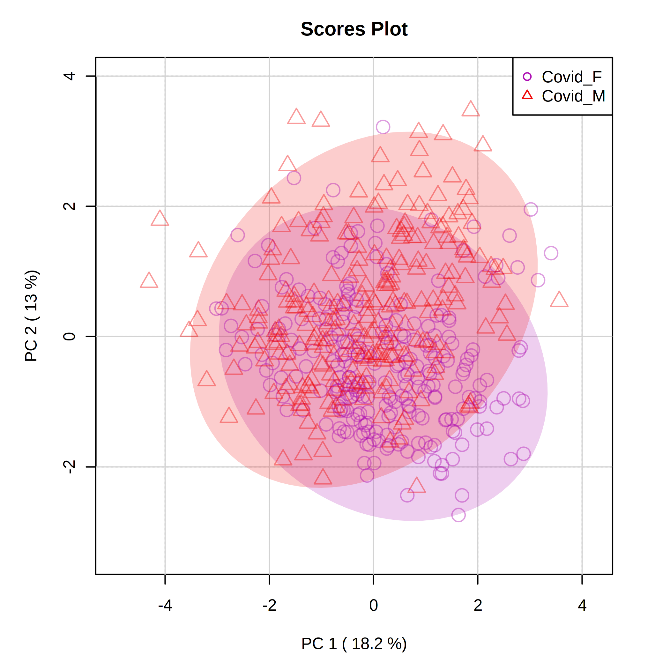

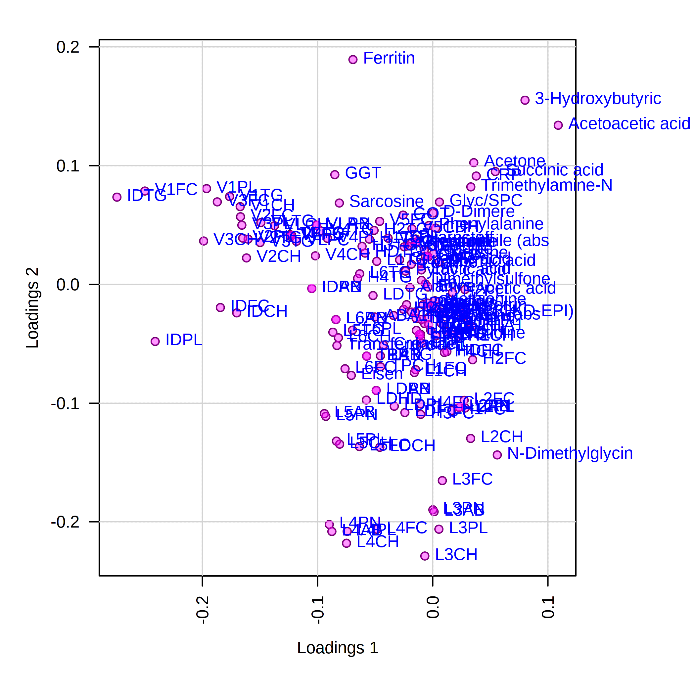

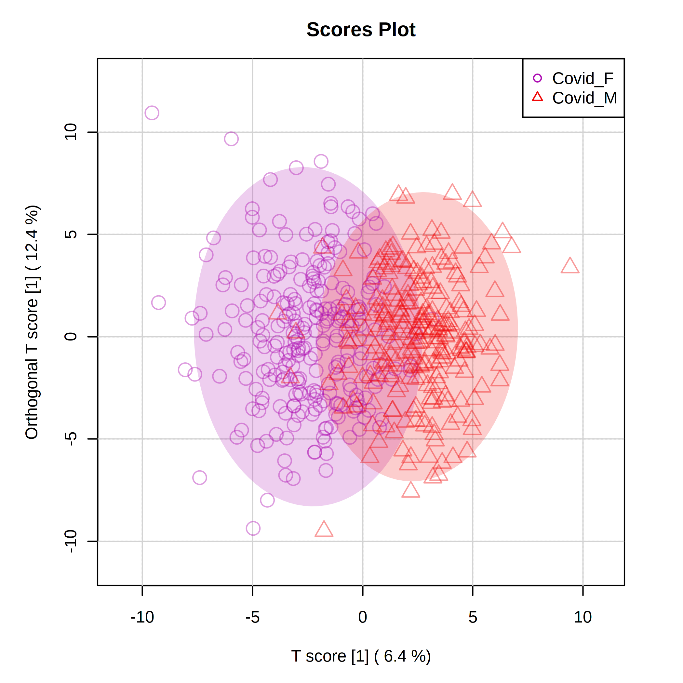

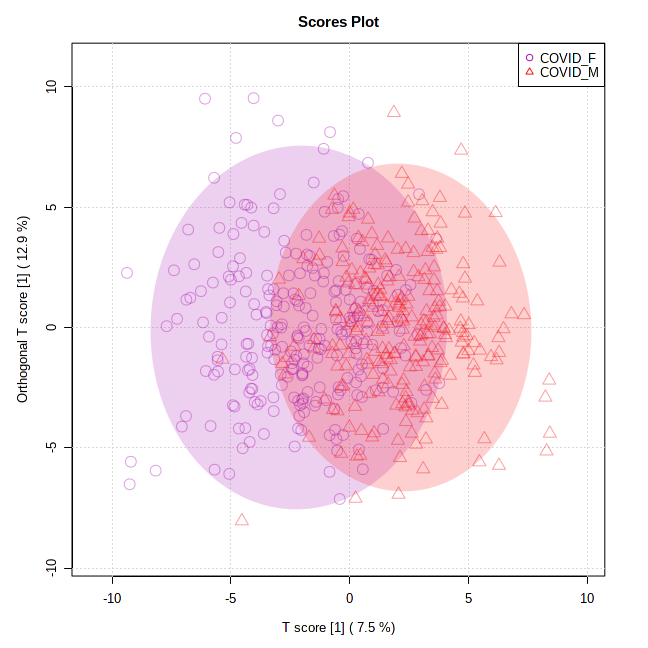

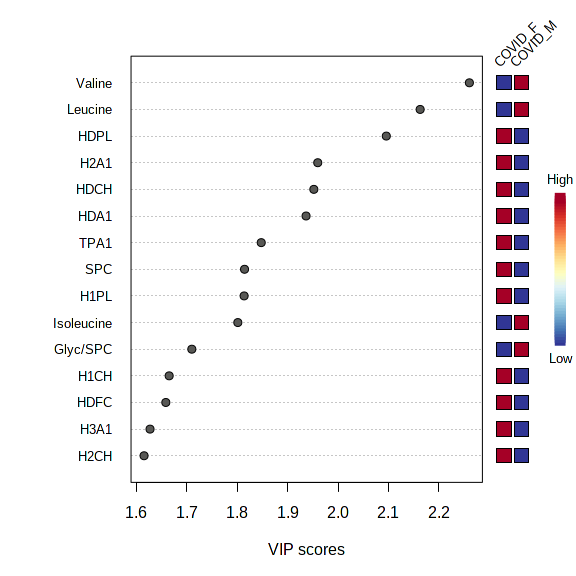


a

b

c

e

f


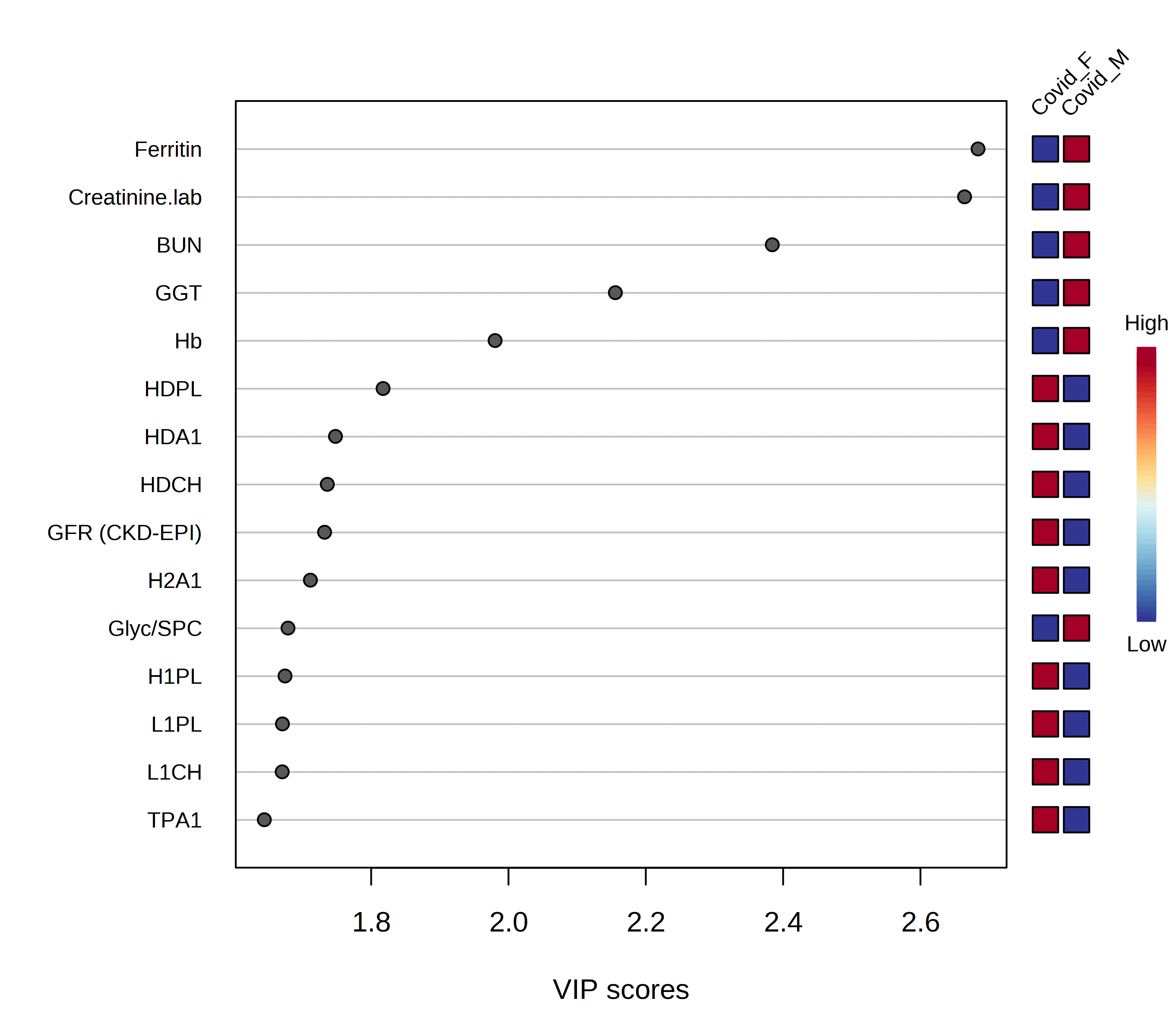


d

***Supplementary Figure 4: Multivariate analysis of the gender comparison of the COVID-19 cohort***

The PCA **(a)** gives an overview of the distribution of female and male COVID-19 patients. Female patients (n=255) are represented by purple circles, male patients by red triangles (n= 254). **(b)** shows the associated loadings plot. The OPLSDA **(c)** illustrates the separation between the gender by orthogonal T scores and T scores. The VIP scores plot **(d)** shows the 15 most important lipoproteins which drive the separation of the two groups. The legend on the right side of the plot in **(d)** specifies if the lipoproteins are high or low in the respective cohort. **(e)** and **(f)** show again an OPLSDA and a VIP scores plot, but with the gender sensitive features creatinine, BUN, ferritin, and liver values, which contribute a large part to the separation of the groups. BUN blood urea nitrogen, F female, GFR glomerular filtration rate, GGT: gamma-glutamate transferase, Glyc glycoprotein, Hb hemoglobin, M male, OPLSDA orthogonal partial least squares- discriminant analysis, PCA principal component analysis, SPC supramolecular phospholipid composite, VIP variable importance in projection.

**Supplementary Figure 5**


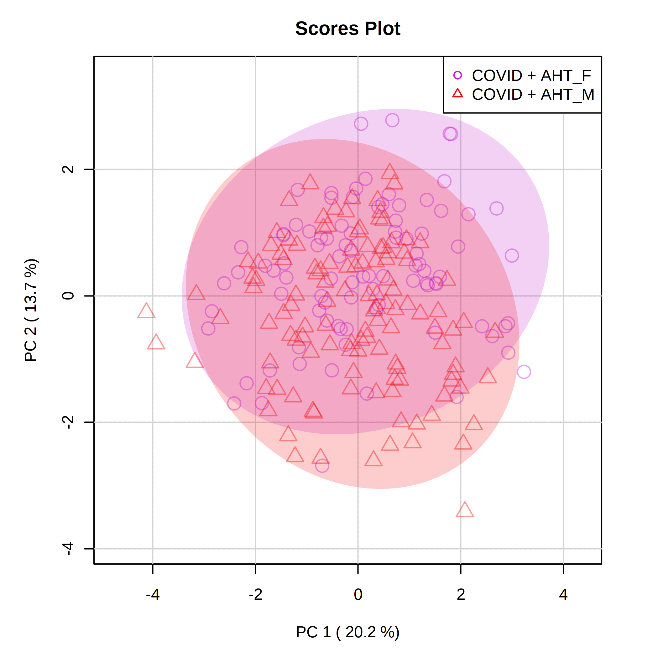

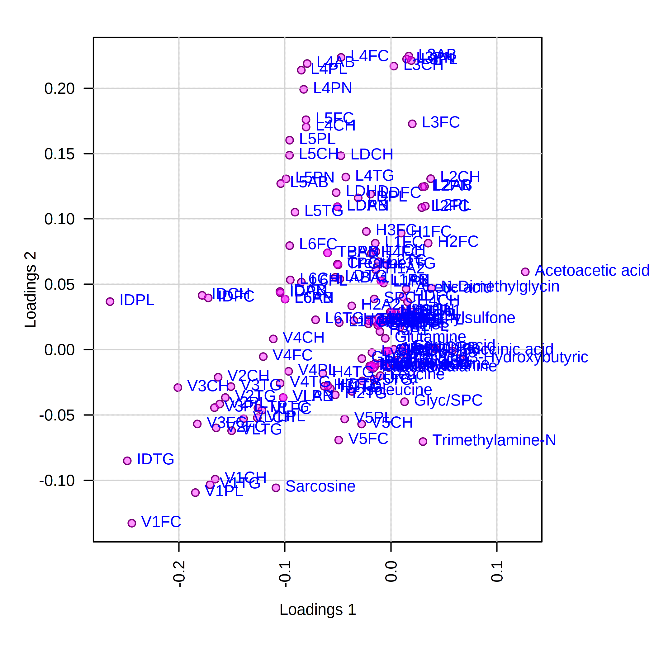

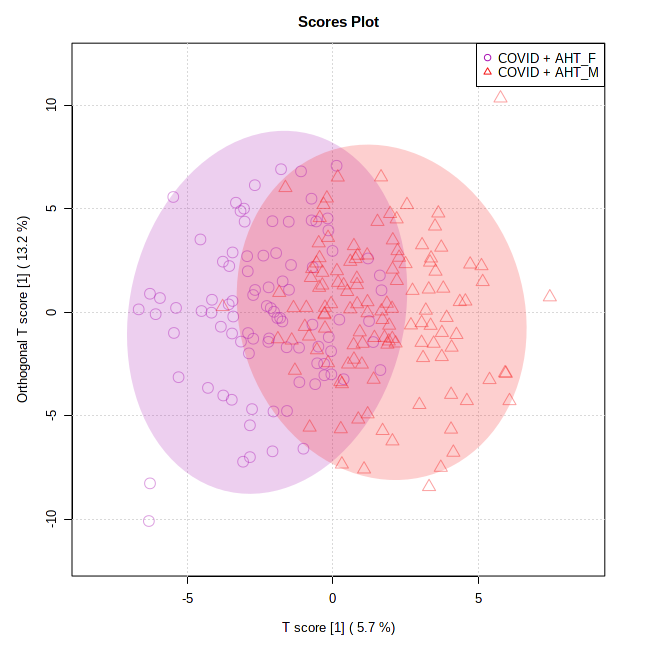


a

b

c

f


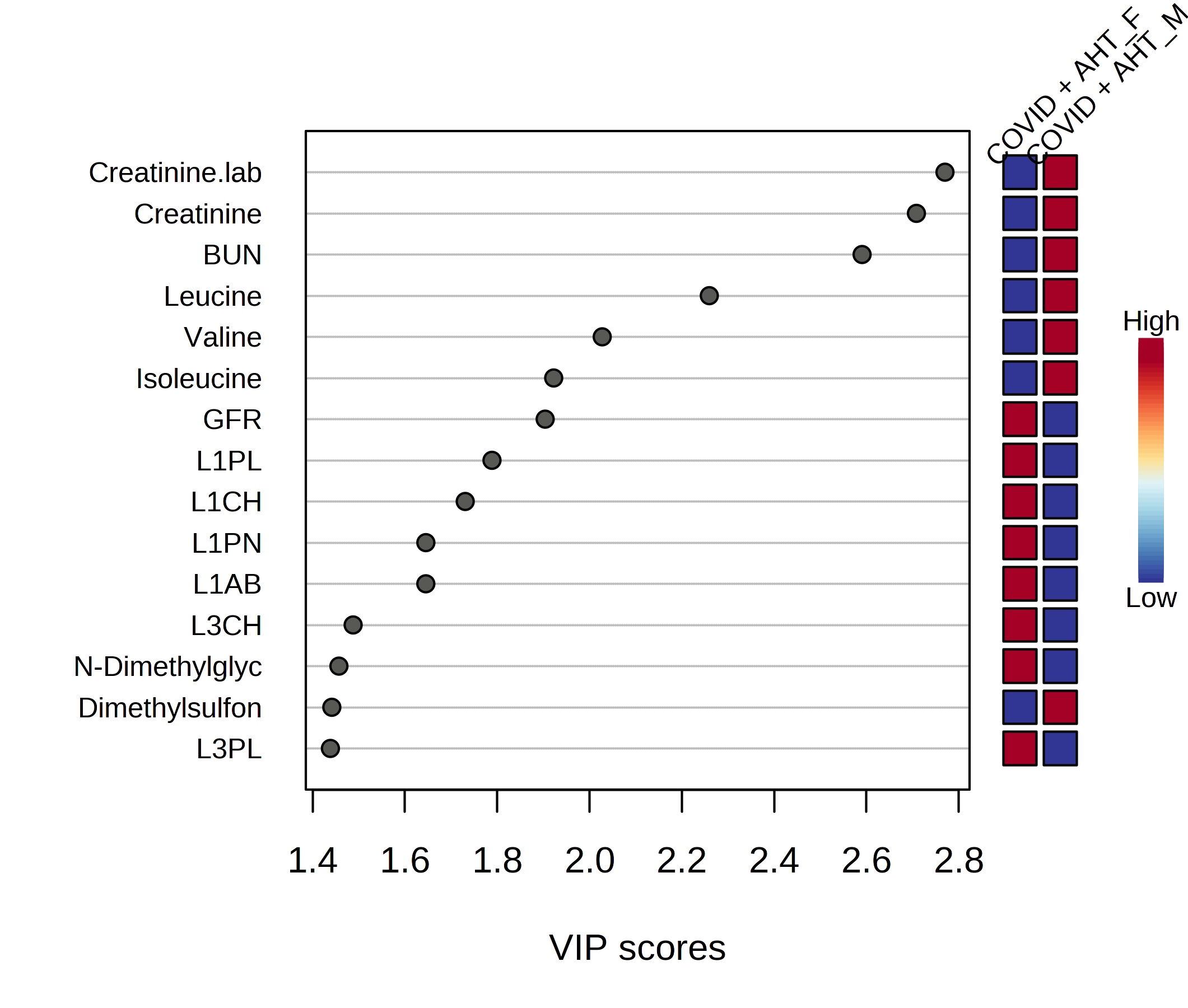


d

**Supplementary Figure 5: Multivariate analysis of the gender comparison of the COVID-19 + AHT cohort**

The PCA **(a)** gives an overview of the distribution of female and male COVID-19 + AHT patients. Female patients (n=92) are represented by purple circles, male patients by red triangles (n= 124). **(b)** shows the associated loadings plot. The OPLSDA **(c)** illustrates the separation between the gender by orthogonal T scores and T scores. The VIP scores plot **(d)** shows the 15 most important lipoproteins which drive the separation of the two groups. The legend on the right side of the plot in **(d)** specifies if the lipoproteins are high or low in the respective cohort. AHT arterial hypertension, BUN blood urea nitrogen, F female, GFR glomerular filtration rate, M male, OPLSDA orthogonal partial least squares- discriminant analysis, PCA principal component analysis, VIP variable importance in projection.

**Supplementary Figure 6**


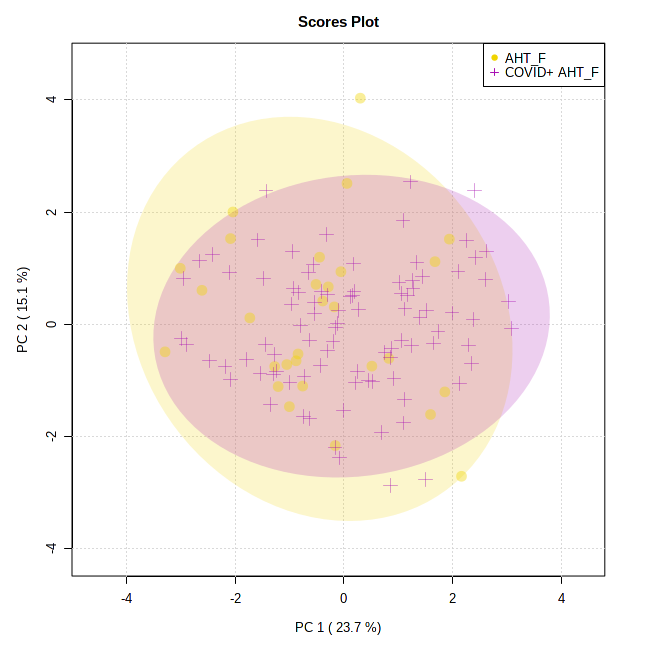

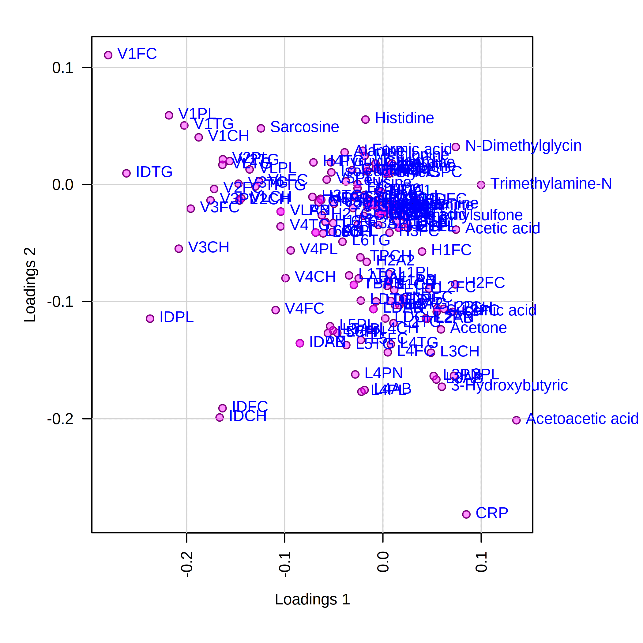

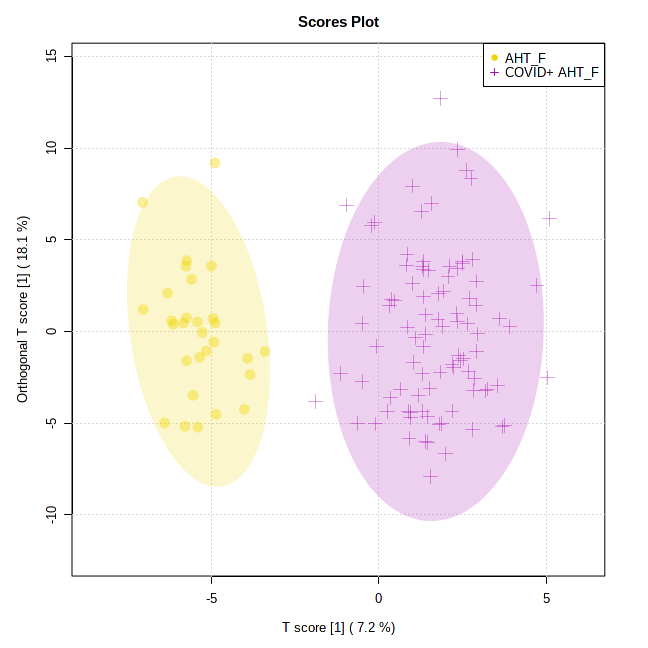

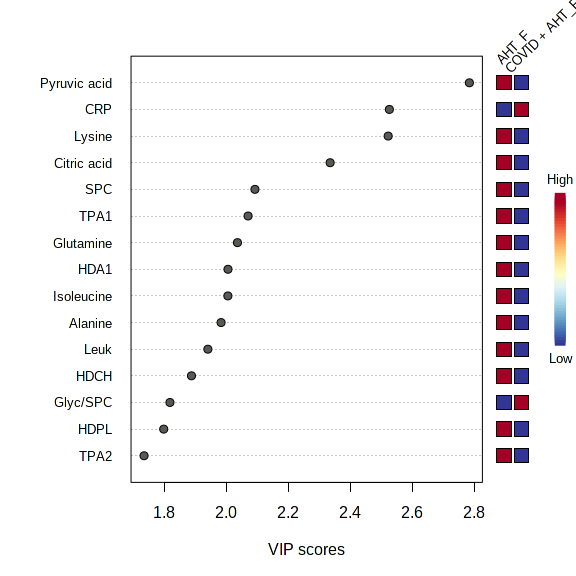


a**~~A~~ A volcano plot was also performed for this and 124 samples from 71 men were compared with 92 samples from 63 women.**

b**~~A~~ A volcano plot was also performed for this and 124 samples from 71 men were compared with 92 samples from 63 women.**

c**~~A~~ A volcano plot was also performed for this and 124 samples from 71 men were compared with 92 samples from 63 women.**

d**~~A~~ A volcano plot was also performed for this and 124 samples from 71 men were compared with 92 samples from 63 women.**

**Supplementary Figure 6: Multivariate Analysis AHT vs. COVID-19 + AHT: Female**

The PCA **(a)** gives an overview of the distribution of female hypertensive COVID-19 patients compared to females from the AHT control cohort. Female COVID-19 + AHT patients (n=92) are represented by purple crosses, female AHT patients without COVID-19 by yellow dots (n= 29). **(b)** shows the associated loadings plot. The OPLSDA **(c)** illustrates the separation between the groups by orthogonal T scores and T scores. The VIP scores plot **(d)** shows the 15 most important lipoproteins which drive the separation of the two groups. The legend on the right side of the plot in **(d)** specifies if the lipoproteins are high or low in the respective cohort. AHT arterial hypertension, CRP C-reactive protein, F female, Glyc glycoprotein, Leuk leukocytes, OPLSDA orthogonal partial least squares- discriminant analysis, PCA principal component analysis, SPC supramolecular phospholipid composite, VIP variable importance in projection.

**Supplementary Figure 7**


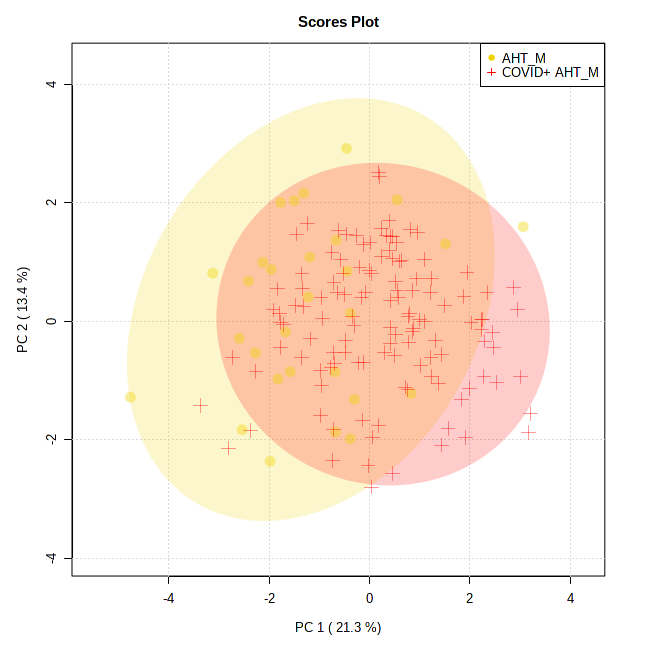

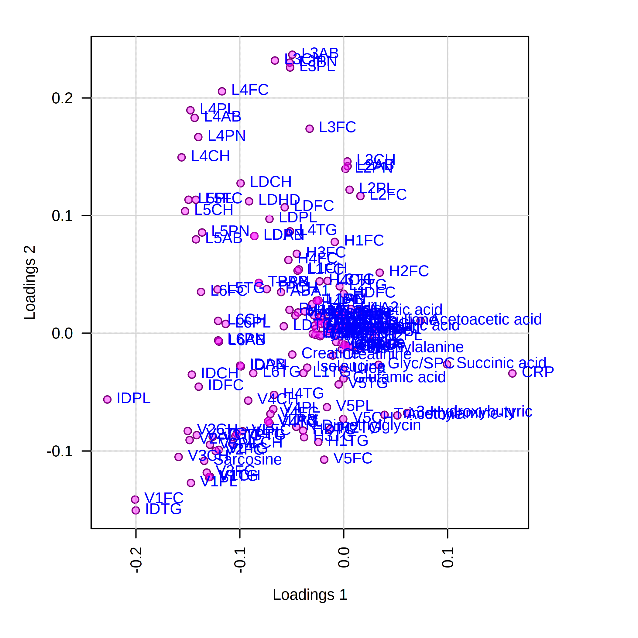

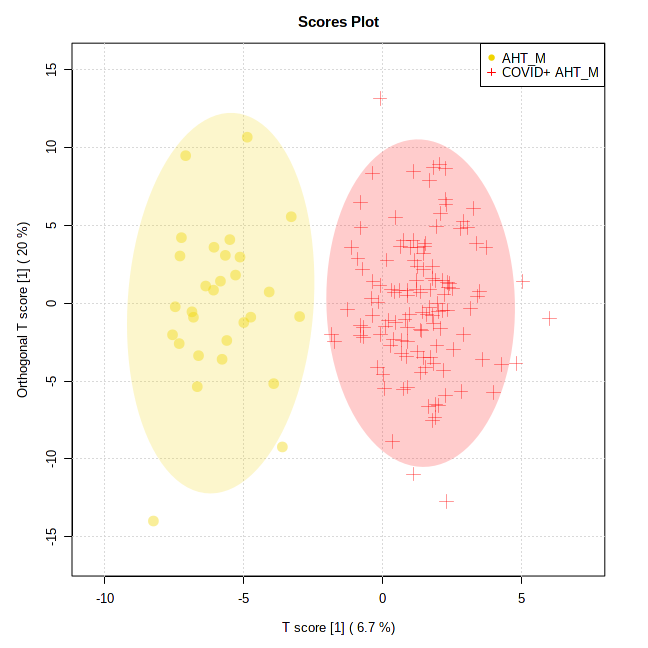

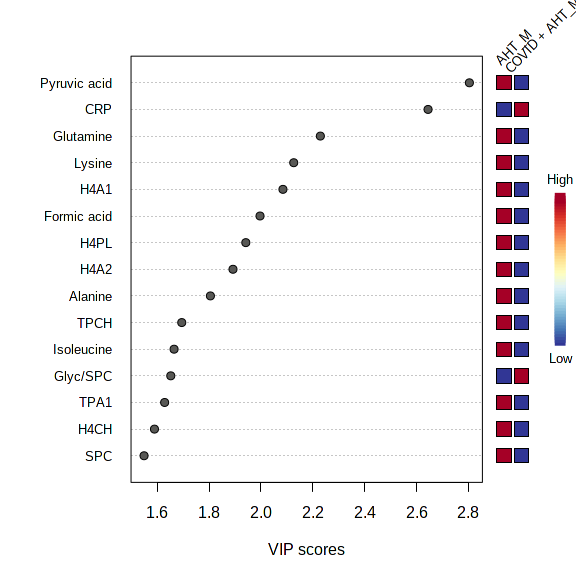


a

b

c

d

**Supplementary Figure 7: Multivariate Analysis AHT vs. COVID-19 + AHT: Male**

The PCA **(a)** gives an overview of the distribution of male hypertensive COVID-19 patients compared to males from the AHT control cohort. Male COVID-19 + AHT patients (n= 124) are represented by red crosses, male AHT patients without COVID-19 by yellow dots (n= 29). **(b)** shows the associated loadings plot. The OPLSDA **(c)** illustrates the separation between the groups by orthogonal T scores and T scores. The VIP scores plot **(d)** shows the 15 most important lipoproteins which drive the separation of the two groups. The legend on the right side of the plot in **(d)** specifies if the lipoproteins are high or low in the respective cohort. AHT arterial hypertension, CRP C-reactive protein, Glyc glycoprotein, M male, OPLSDA orthogonal partial least squares- discriminant analysis, PCA principal component analysis, SPC supramolecular phospholipid composite, VIP variable importance in projection.

**Supplementary Figure 8**


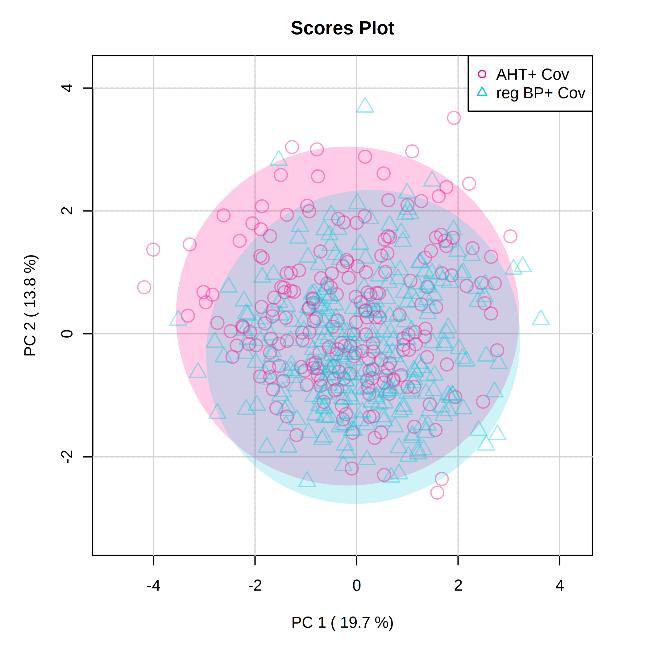

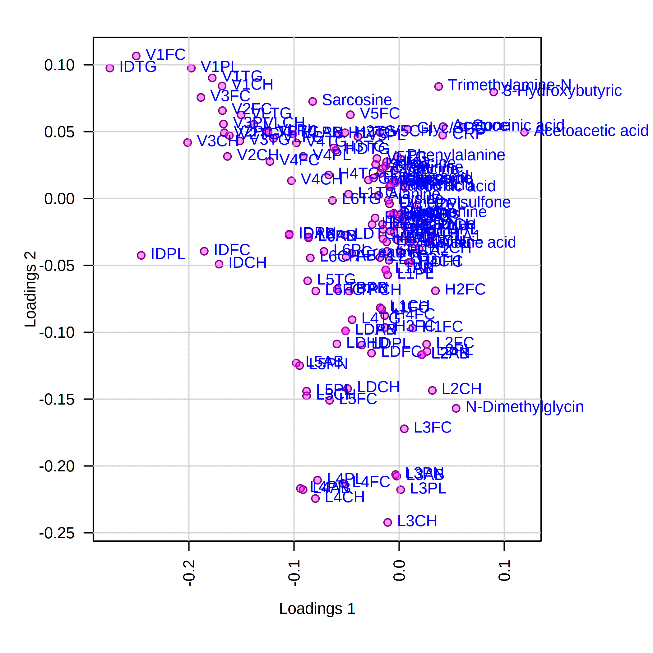

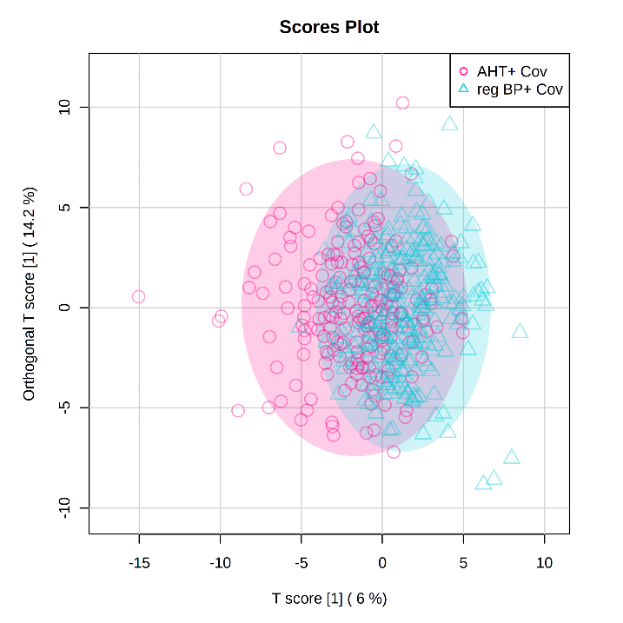


a

b

c

d~~a~~


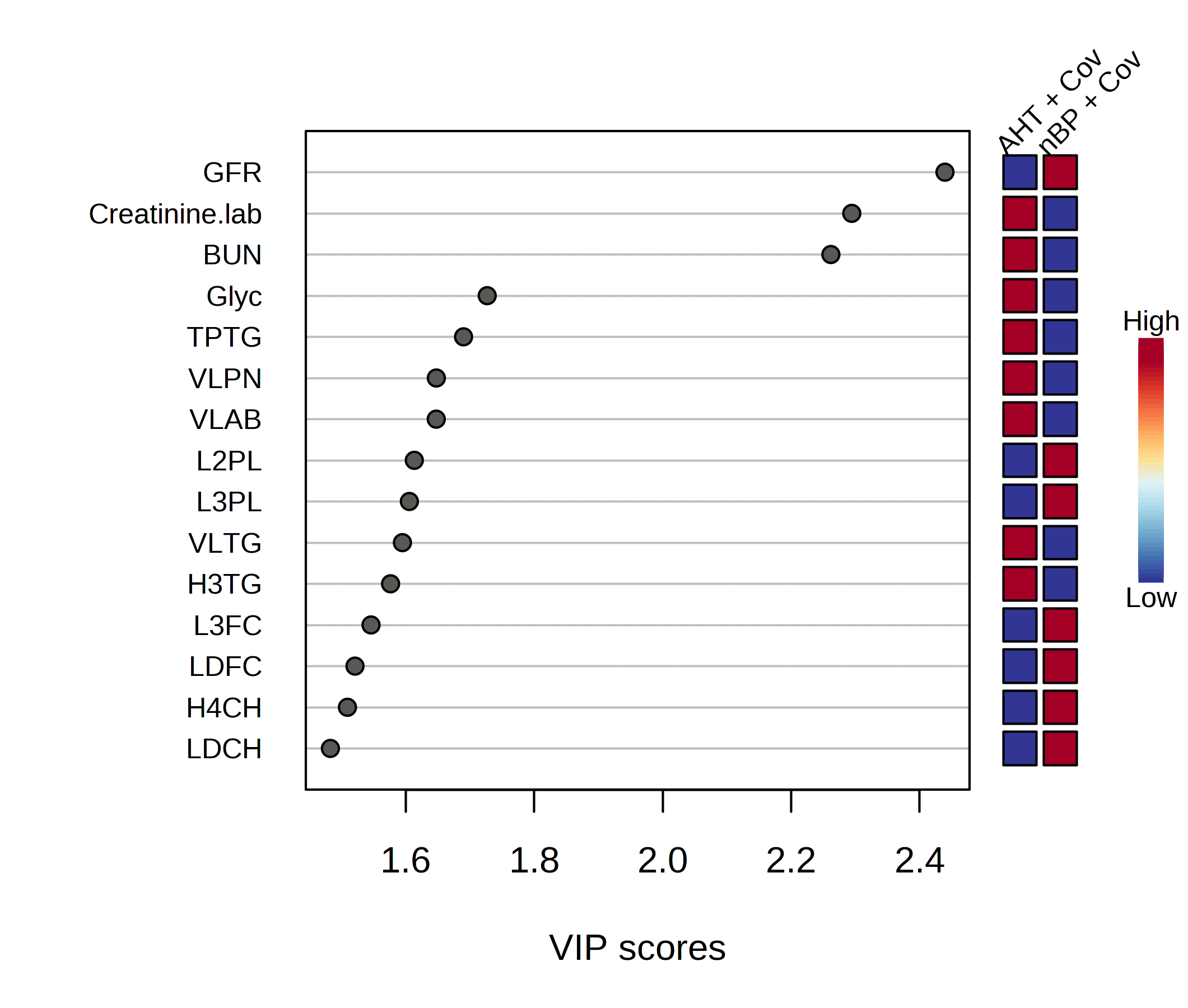


d

**Supplementary Figure 8: Multivariate analysis of COVID-19 with AHT versus regular BP**

The PCA **(a)** gives an overview of the distribution of the hypertensive COVID-19 patients (n= 216) with pink circles, and COVID-19 patients with regular blood pressure (n= 293) with blue triangles. **(b)** shows the associated loadings plot. The OPLSDA **(c)** illustrates the separation between the groups by orthogonal T scores and T scores. The VIP scores plot **(d)** shows the 15 most important lipoproteins which drive the separation of the two groups. The legend on the right side of the plot in **(d)** specifies if the lipoproteins are high or low in the respective cohort. AHT arterial hypertension, BP blood pressure, BUN blood urea nitrogen, Cov COVID-19, GFR glomerular filtration rate, OPLSDA orthogonal partial least squares- discriminant analysis, PCA principal component analysis, VIP variable importance in projection.

**Supplementary Figure 9**


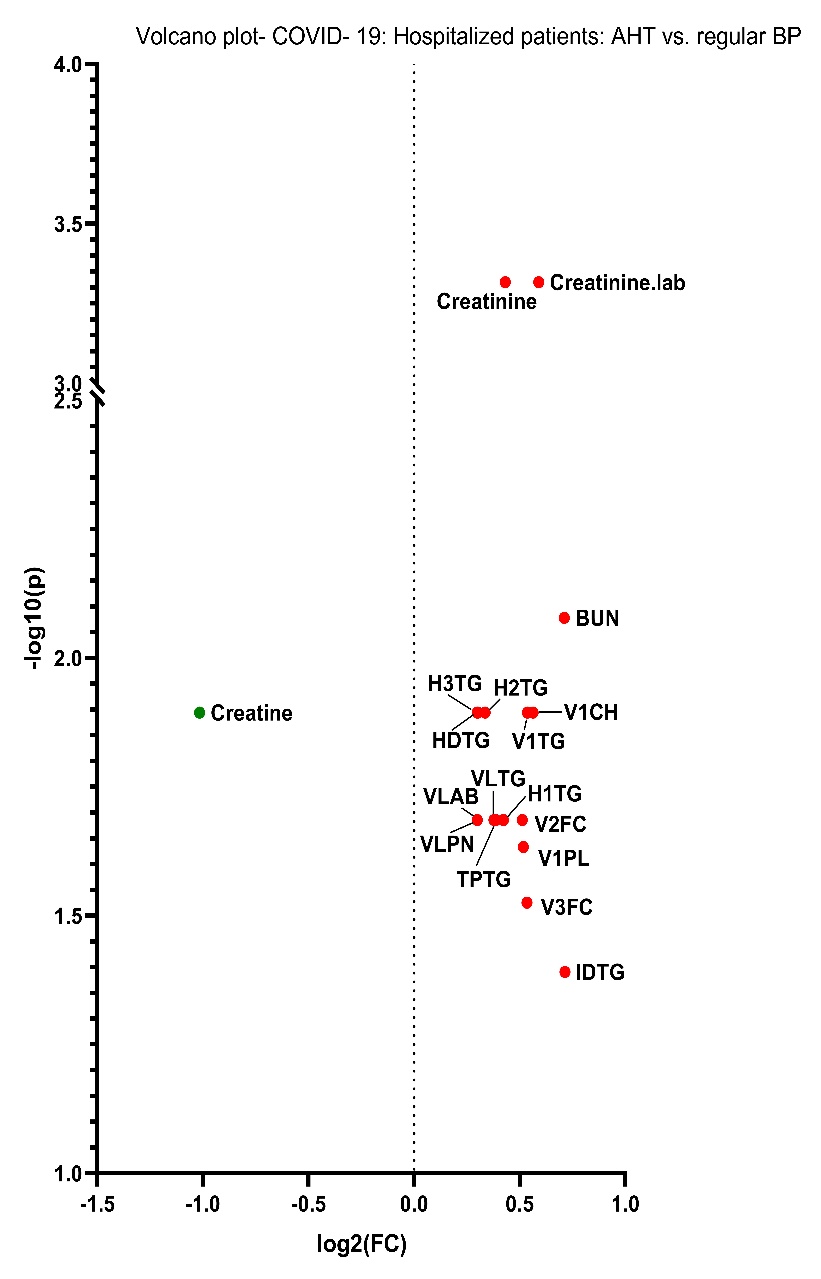


a


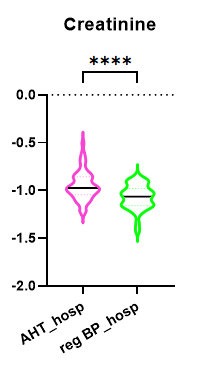


b


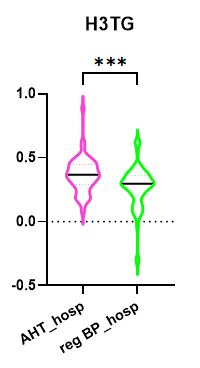

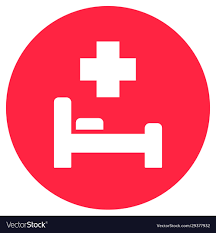

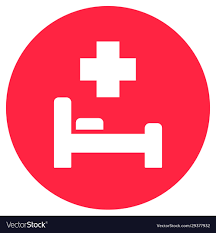

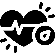


**Supplementary Figure 9: Metabolomic characteristics of hospitalized COVID-19 patients with AHT**

In **(a)** univariate analysis by means of volcano plot showing significant differences between normotensive (n= 71) and hypertensive (n= 56) hospitalized COVID-19 patients (FC > 1.2, p < 0.05, FDR < 0.01): The red points show increased features in the hospitalized COVID-19 + AHT cohort, while the green one is decreased. In **(b)** violin plots with max. to min., whisker, and median, show the most significant alterations of the NMR parameter Creatinine and H3TG. The y-axis shows the normalized concentration. AHT arterial hypertension, BP blood pressure, BUN blood urea nitrogen, Creatinine.lab creatinine from laboratory report, hosp hospitalized.

**Supplementary Figure 10**


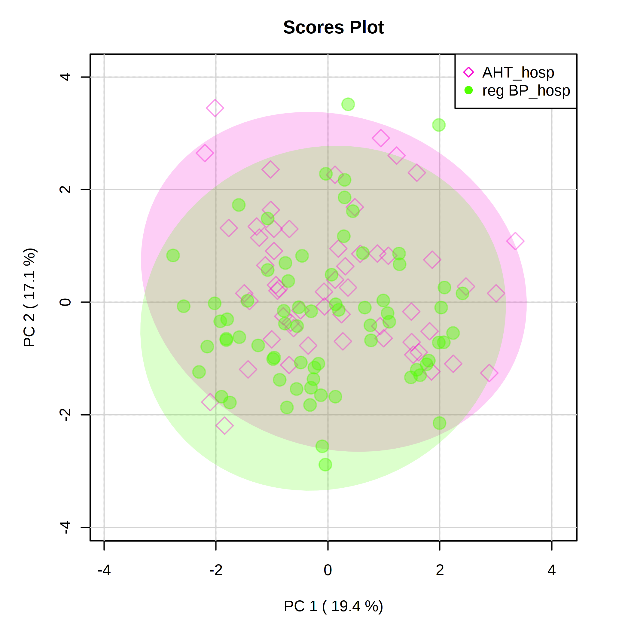

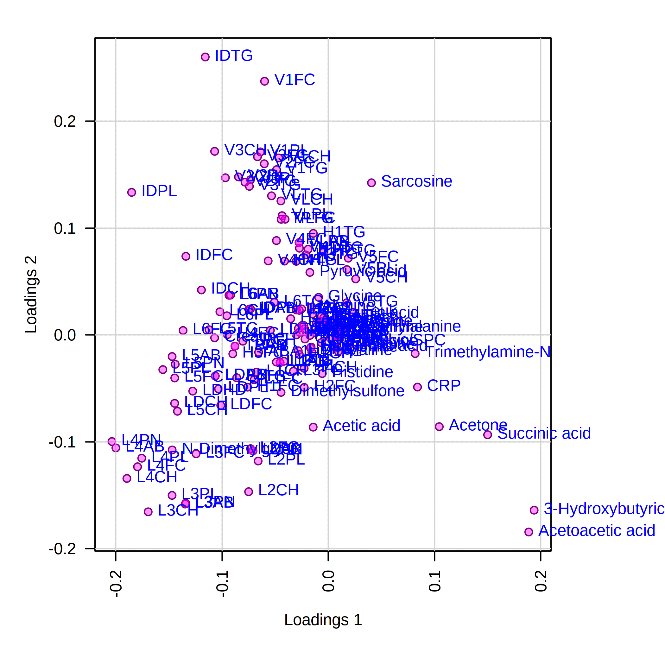


a~~A~~

b


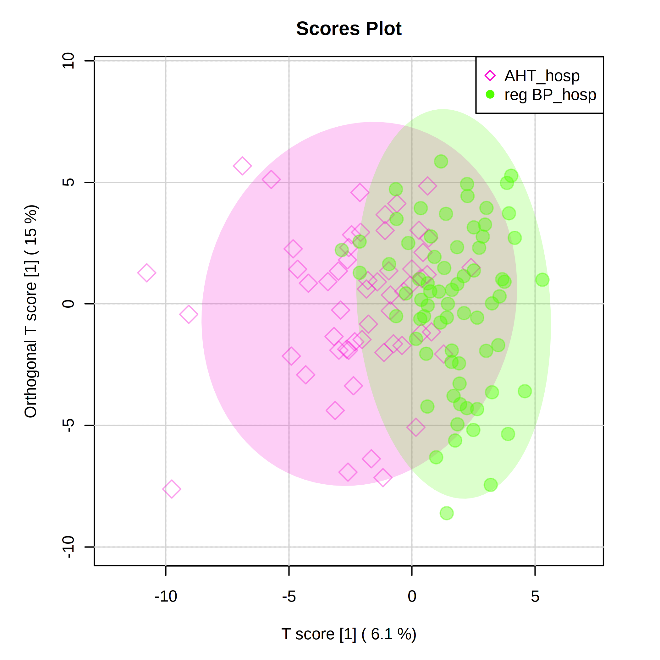


c~~a~~


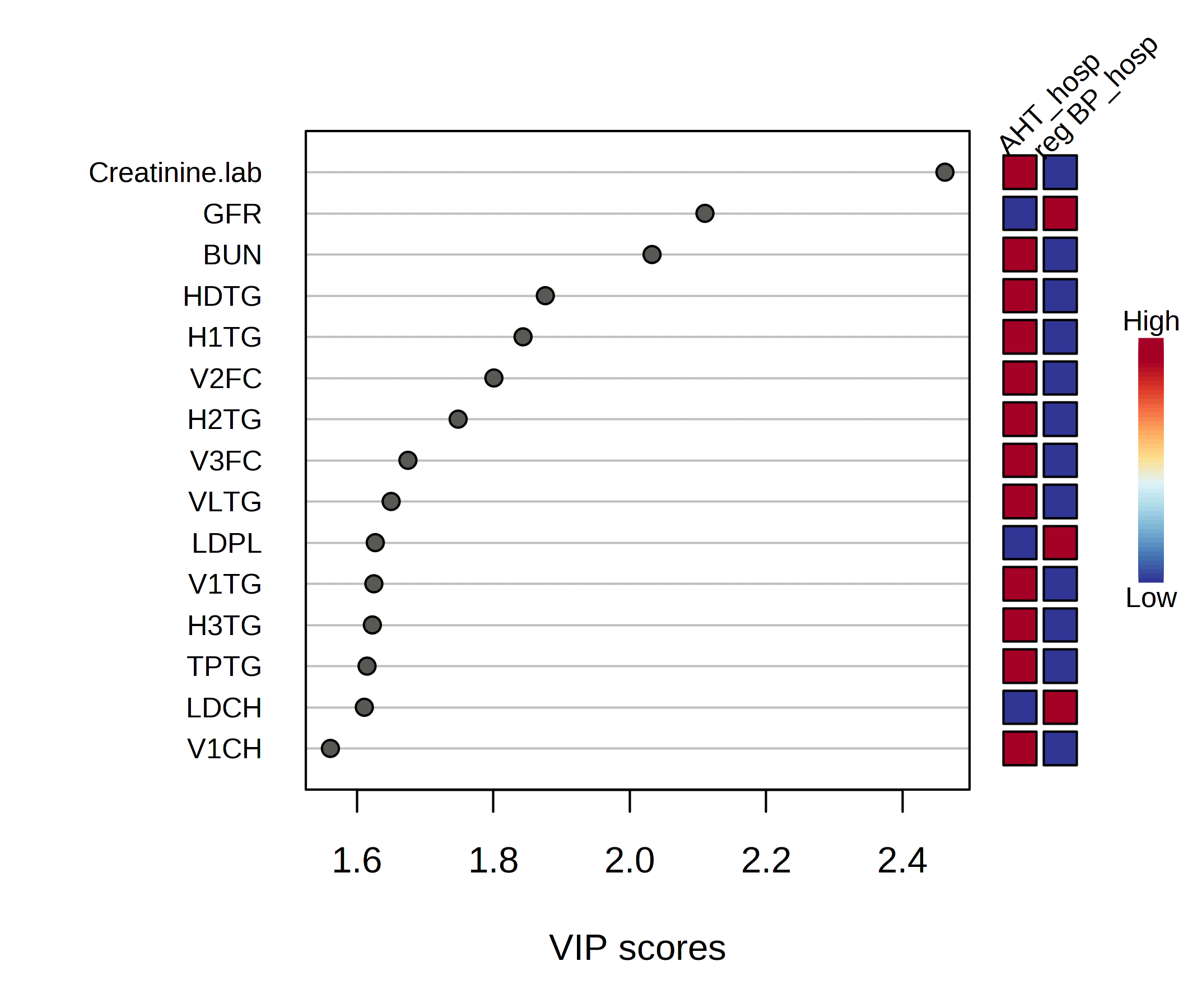


d~~a~~

**Supplementary Figure 10: Multivariate analysis of hospitalized COVID-19 with AHT versus regular BP**

The PCA **(a)** gives an overview of the distribution of the hospitalized COVID-19 patients with AHT (n= 56), represented by pink rhombuses, and hospitalized COVID-19 patients with regular blood pressure (n= 71), represented by green rhombuses. **(b)** shows the associated loadings plot. The OPLSDA **(c)** illustrates the separation between the groups by orthogonal T scores and T scores. The VIP scores plot **(d)** shows the 15 most important lipoproteins which drive the separation of the two groups. The legend on the right side of the plot in **(d)** specifies if the lipoproteins are high or low in the respective cohort. AHT arterial hypertension, BP blood pressure, BUN blood urea nitrogen, GFR glomerular filtration rate, hosp hospitalized, OPLSDA orthogonal partial least squares- discriminant analysis, PCA principal component analysis, VIP variable importance in projection.

**Supplementary Figure 11**


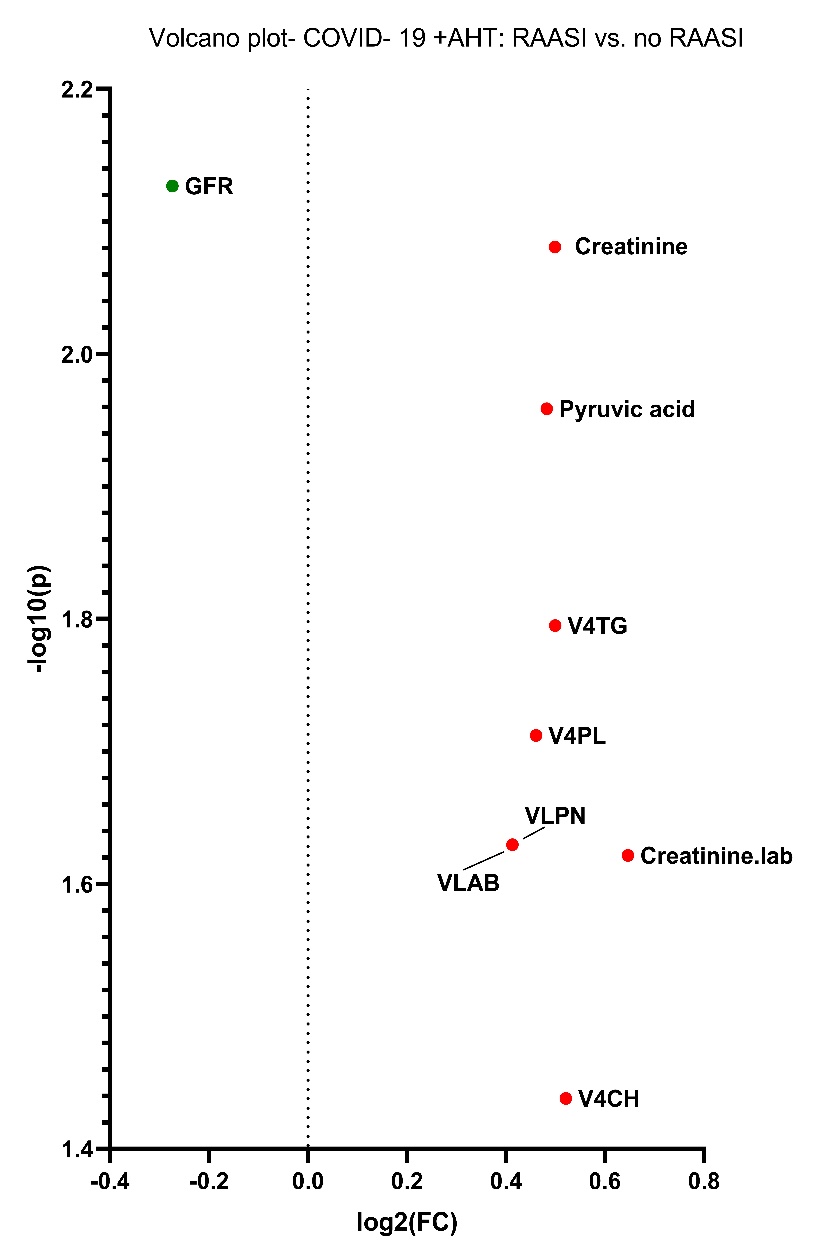

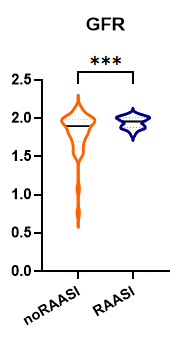

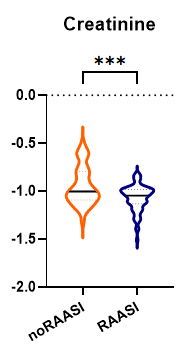


b

a

**Supplementary Figure 11: Hypertensive COVID-19 patients, separated in two antihypertensive treatment groups: Renin- angiotensin-aldosterone system inhibitors versus other (beta-blockers, calcium channel blockers)**

In **(a)** univariate comparison by means of volcano plot analysis (FC > 1.2, p < 0.05, FDR < 0.01): The red features correspond to increased values in the RAASI group (n= 75, no RAASI: n= 40), while the green ones correspond to decreased values. **(b)** Violin plots with max. to min., whisker, and median, show significant alterations in the kidney parameters creatinine and GFR. The y-axis shows the normalized concentration. Creatinine.lab creatinine from laboratory report, GFR glomerular filtration rate.

**Supplementary Figure 12**


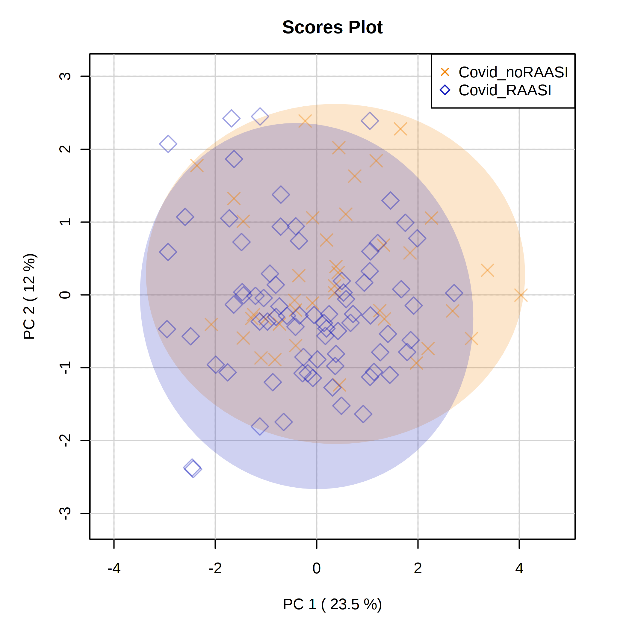

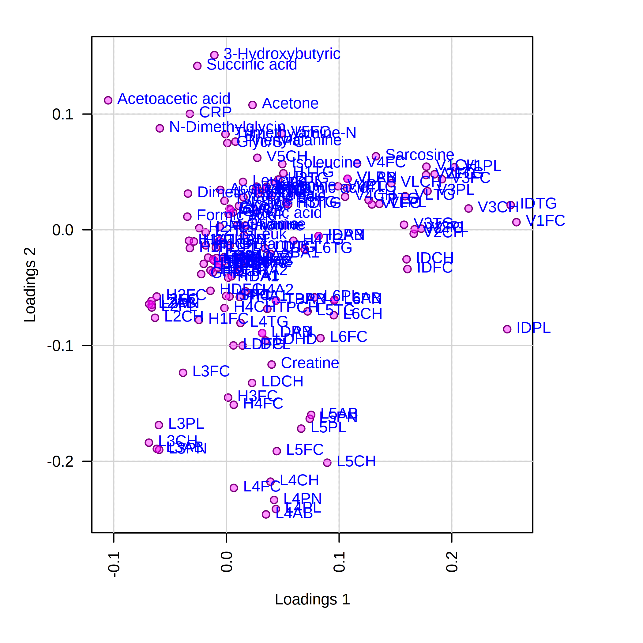

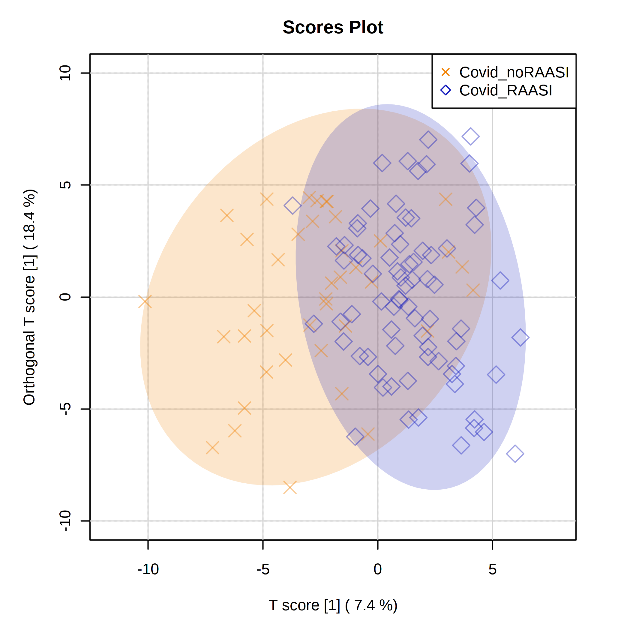


a

b

c


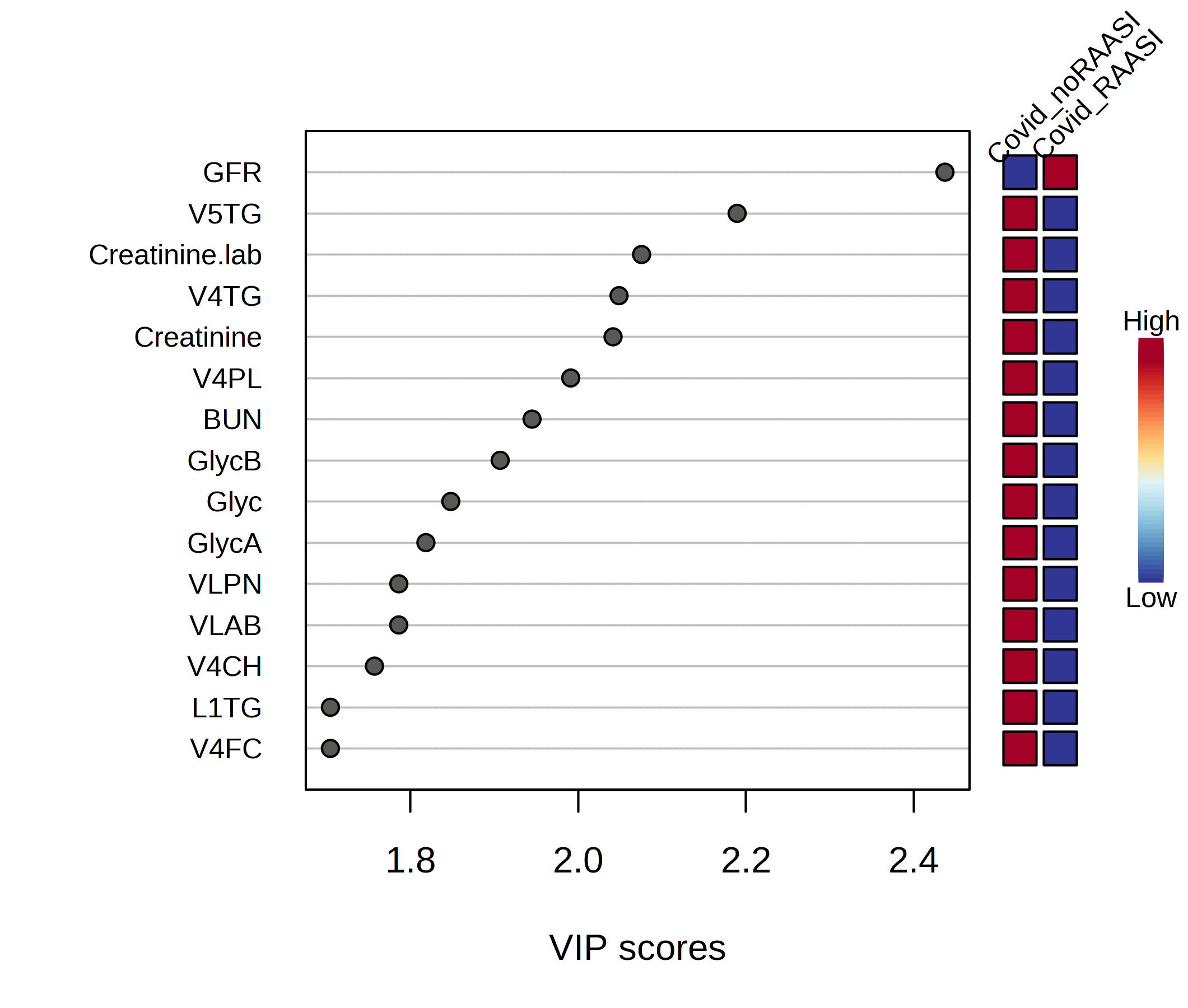


d~~a~~

**Supplementary Figure 12: Multivariate analysis of hypertensive COVID-19 patients, separated in two antihypertensive treatment groups: Renin- angiotensin-aldosterone system inhibitors (RAASI) versus other (beta-blockers, calcium channel blockers)**

The PCA **(a)** gives an overview of the distribution of the COVID-19 + AHT cohort, separated in the RAASI group (n= 75), represented by blue rhombuses, and the no RAASI group, represented by yellow crosses (n= 40). **(b)** shows the associated loadings plot. The OPLSDA **(c)** illustrates the separation between the groups by orthogonal T scores and T scores. The VIP scores plot **(d)** shows the 15 most important lipoproteins which drive the separation of the two groups. The legend on the right side of the plot in **(d)** specifies if the lipoproteins are high or low in the respective cohort. BUN blood urea nitrogen, GFR glomerular filtration rate, Glyc glycoprotein, OPLSDA orthogonal partial least squares- discriminant analysis, PCA principal component analysis, RAASI: Renin-angiotensin-aldosterone system inhibitors, VIP variable importance in projection.

**Supplementary Figure 13**


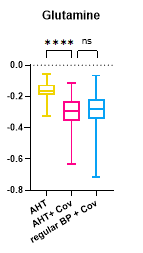

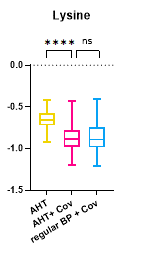

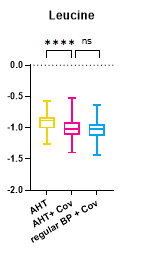

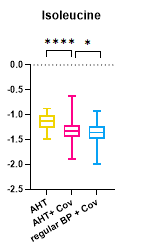

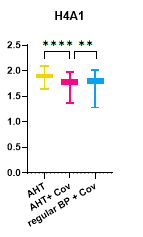

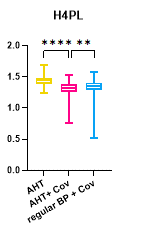

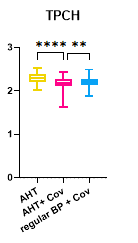


a

b

c

d

e

f

g

**Supplementary Figure 13: Characteristic metabolites and lipoproteins in the serum of COVID-19 patients**

Box plots with max. to min., whisker, and median, showing typical altered NMR parameters in hypertensive (pink, n= 216) and normotensive (blue, n=293) COVID-19 patients, and in the AHT control cohort (yellow, n= 58): Glutamine (a), lysine (b), leucine (c), isoleucine (d), H4A1 (e), H4PL (f), and TPCH (g). Unpaired t-tests were performed for the comparison of AHT versus COVID-19 + AHT, and COVID-19 with and without AHT. Significant alterations are shown in form of asterisks: ns (not significant), p < 0.05 (*), p < 0.01 ( ⃰ ⃰ ), p < 0.001 ( ⃰ ⃰ ⃰ ), p < 0.0001 ( ⃰ ⃰ ⃰ ⃰ ). The y-axis shows the normalized concentration. AHT arterial hypertension, BP blood pressure, Cov COVID-19.

**Supplementary Figure 14**


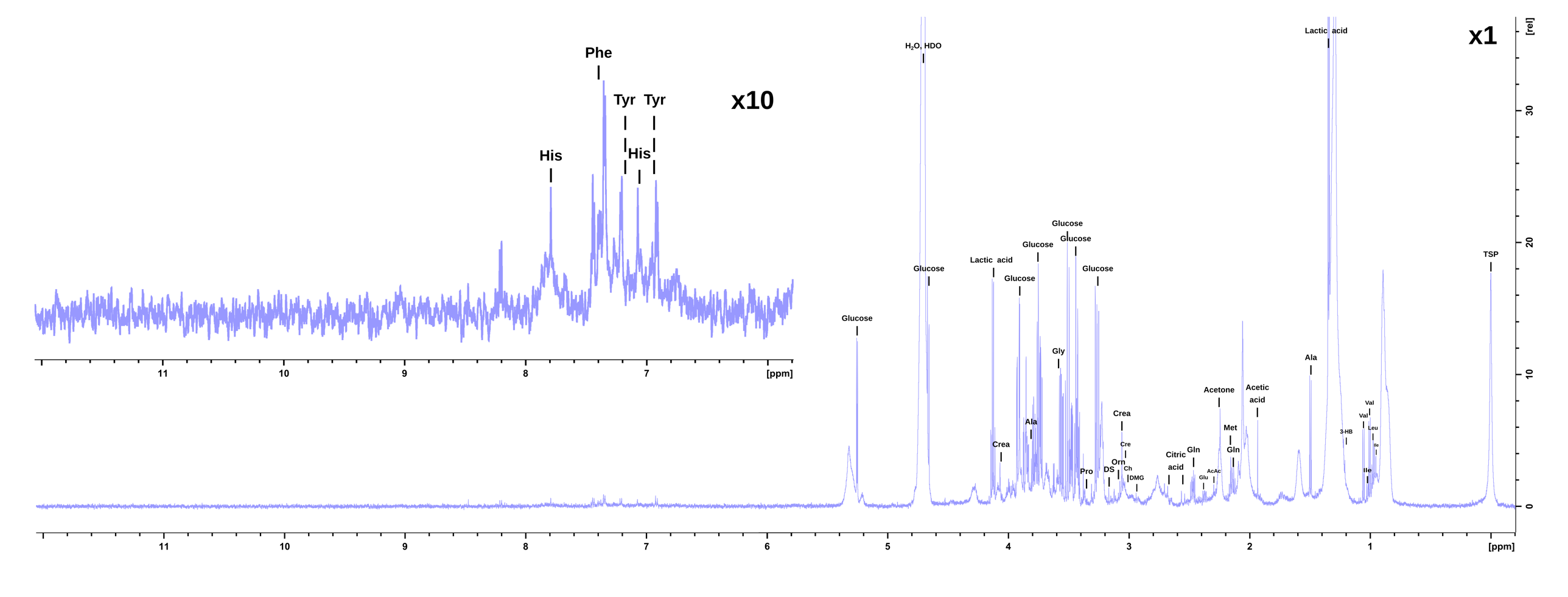




**a**

**b**

**Supplementary Figure 14: Exemplary 1D ^1^H NMR spectra of a blood serum sample used to analyze polar metabolites, glycoproteins and SPC:**

*Panel a illustrates a CPMG spectrum of quantified metabolites. The aromatic region (8.0 to 6.0 ppm) is zoomed by factor 10. HDO semi heavy water, Crea creatinine, DS dimethylsulfone, Cre creatine, Ch choline, DMG N, N-dimethylglycine, AcAc acetoacetic acid, 3-HB 3-hydroxybutyric acid, Val valine, Ile isoleucine, Leu leucine, TSP 3-(trimethylsilyl) propionic-2,2,3,3-d4 acid.*

Panel b displays a PGPE spectrum of glycoproteins and SPC as new inflammatory NMR parameter. HDO semi heavy water, SPC supramolecular phospholipid composite signal, GlycA glycoprotein A, GlycB glycoprotein B.
