# Supplementary material for: Stratification of hypertension and SARS-CoV-2 infection by quantitative NMR spectroscopy of human blood serum
Source: Commun Med (Lond). 2023 Oct 16;3:145. doi: 10.1038/s43856-023-00365-y (PMC11081957; doi:10.1038/s43856-023-00365-y)
Supplement: Supplementary file 3 — Description of Supplementary Materials [file 43856_2023_365_MOESM3_ESM.docx]

Supplementary Data 1: List of abbreviations of all 112 lipoprotein parameters with their units, which can be analyzed with the B.I.LISA^TM^ package from Bruker BioSpin GmbH.

Supplementary Data 2: These tables show the data from all volcano plot analyses, a combination of unpaired t-test and fold change analysis, which led to Figures 2, 3, and 4, and to Supplementary Figures 3,9, and 11. The data are sorted by p-value. The abbreviations for all lipoproteins can be seen in the Supplementary Data 1. FC fold change, p p-value.

Supplementary Data 3: NMR data from the B.I.LISA^TM^ , B.I. PACS ^TM,^ , and B.I. Quant-PS ^TM^ reports for the COVID-19 cohort, AHT control cohort, and the Healthy control (HC) cohort from Buker BioSpin GmbH. Routine laboratory parameters were available from the COVID-19 cohort, and the AHT control cohort.

Supplementary Data 4: Clinical metadata of the AHT control cohort. Listed are age range, gender, diabetes mellitus as comorbidity, antihypertensive treatment, and the dates of study inclusion and sample collection.

Supplementary Data 5: Clinical metadata of the COVID-19 cohort. The tables contain information about general characteristics, diseases severity criteria, vital signs, pre-existing disorders, and pre-medication. From these data, Tables 1 and 2 of the main manuscript were prepared, as well as Supplementary Tables 3, 4, 5 and 6.
