# Supplementary material for: Stratification of hypertension and SARS-CoV-2 infection by quantitative NMR spectroscopy of human blood serum
Source: Commun Med (Lond). 2023 Oct 16;3:145. doi: 10.1038/s43856-023-00365-y (PMC11081957; doi:10.1038/s43856-023-00365-y)
Supplement: Supplementary file 9 — Reporting Summary [file 43856_2023_365_MOESM9_ESM.pdf]

nature portfolio

Corresponding author(s): Dr. Christoph Trautwein

Last updated by author(s): 16/6/23

Reporting Summary

Nature Portfolio wishes to improve the reproducibility of the work that we publish. This form provides structure for consistency and transparency in reporting. For further information on Nature Portfolio policies, see our Editorial Policies and the Editorial Policy Checklist.

Statistics

For all statistical analyses, confirm that the following items are present in the figure legend, table legend, main text, or Methods section.

n/a	Confirmed	
☐	☒	The exact sample size (*n*) for each experimental group/condition, given as a discrete number and unit of measurement
☐	☒	A statement on whether measurements were taken from distinct samples or whether the same sample was measured repeatedly
☐	☒	The statistical test(s) used AND whether they are one- or two-sided<br>*Only common tests should be described solely by name; describe more complex techniques in the Methods section.*
☒	☐	A description of all covariates tested
☐	☒	A description of any assumptions or corrections, such as tests of normality and adjustment for multiple comparisons
☐	☒	A full description of the statistical parameters including central tendency (e.g. means) or other basic estimates (e.g. regression coefficient) AND variation (e.g. standard deviation) or associated estimates of uncertainty (e.g. confidence intervals)
☒	☐	For null hypothesis testing, the test statistic (e.g. *F*, *t*, *r*) with confidence intervals, effect sizes, degrees of freedom and *P* value noted<br>*Give P values as exact values whenever suitable.*
☒	☐	For Bayesian analysis, information on the choice of priors and Markov chain Monte Carlo settings
☒	☐	For hierarchical and complex designs, identification of the appropriate level for tests and full reporting of outcomes
☒	☐	Estimates of effect sizes (e.g. Cohen's *d*, Pearson's *r*), indicating how they were calculated

Our web collection on statistics for biologists contains articles on many of the points above.

Software and code

Policy information about availability of computer code

Data collection	The NMR spectra were acquired using Bruker's body fluids NMR methods package [https://www.bruker.com/de/products-and-solutions/mr/nmr-clinical-research-solutions/b-i-methods.html]. The spectra (NOESYs) were analyzed using Bruker`s software TopSpin (Version 3.6.2). For collection of the NMR data and preparation for data analysis the following commercial available IVDr SOPs (standard operation procedures) were aquired: B.I.BioBankQC, B.I.Quant-PS™, B.I.LISA™, and B.I. PACS™. All SOPs were provided by Bruker BioSpin GmbH, Ettlingen, Germany.
Data analysis	All Data was analyzed with the open source software package Metaboanalyst 5.0. For better imaging and univariate statistic tests by means of unpaired t–tests GraphPad Prism 9 was used.

For manuscripts utilizing custom algorithms or software that are central to the research but not yet described in published literature, software must be made available to editors and reviewers. We strongly encourage code deposition in a community repository (e.g. GitHub). See the Nature Portfolio guidelines for submitting code & software for further information.

Data

Policy information about availability of data

All manuscripts must include a data availability statement. This statement should provide the following information, where applicable:

- Accession codes, unique identifiers, or web links for publicly available datasets
- A description of any restrictions on data availability
- For clinical datasets or third party data, please ensure that the statement adheres to our policy

Supplementary Data 2 contains the volcano plot analyses data for the Figures 2 a, 2 b, 3 a, 3 b, and 4 from the main manuscript, and for the volcano plots in the Supplementary Figures 3 a, 3 b, 9, and 11. For Figure 5, and for the Supplementary Figures 1, 2, 4, 5, 6, 7, 8, 10, 12, and 13, the NMR Data from the Supplementary Data 3 were divided into subgroups for the statistical comparisons using the available metadata for the AHT control cohort (Supplementary Data 4), and the COVID-19 cohort (Supplementary Data 5). Data reports from the applied commercial panels B.I.BioBank-QC™, B.I.Quant-PS™, B.I.LISA™ and B.I. PACS™ are available upon request.

Human research participants

Policy information about studies involving human research participants and Sex and Gender in Research.

Reporting on sex and gender	One aim of this study was to investigate sex differences in the metabolic profil of COVID-19 disease. Therefore, special attention was given to sex in the COVID-19 and the control cohorts. We compared NMR data of 174 female and 155 male COVID-19 patients. Furthermore, we divided the sexes according to the previous disease arterial hypertension (AHT), for comparing 63 female with 71 male COVID-19 patients with AHT. The AHT control cohort consisted of 29 female and 29 male individuals. This cohort was used to compare hypertensive patients with and without COVID-19 disease separated by sex, respectively.
Population characteristics	The COVID-19 cohort consisted of 329 study participants. Among them were 174 female and 155 male patients. The median age was 54 years with an interquartile range from 44 to 64 years. From the 329 COVID-19 affected, 71 patients needed hospital treatment. Eight of them were treated in the intensive care unit. Five study participants died during study period. 134 COVID-19 affected suffered from arterial hypertension (AHT). Other common pre-condtions included diabetes mellitus (44 patients), coronary artery disease (33 patients), chronic obstructive pulmonary disease (12 patients), asthma (44 patients), and hypothyroidism (46 patients). The AHT control cohort consisted of 58 individuals with 29 females and 29 males, respectively. The median age was 57 years with an interquartile range from 52 to 61 years. As pre- condition only diabetes mellitus was recorded, which affected 22 of the 58 individuals in the AHT control cohort.
Recruitment	The COVID-19 study population consisted of patients monitored with the "Coronataxi digital early warning" (CDEW) system deployed in Rhein-Neckar County and Heidelberg, Germany - an outpatient care system consisting of remote digital monitoring via a mobile application, a medical doctor dashboard and medical care delivery to COVID-19 patients in home quarantine when indicated. The sample collection took place during home visits or in hospital. This CDEW system was part of a study, which was performed by the Universitiy Hospital Heidelberg, Germany. Patients diagnosed with COVID-19 were informed about the opportunity to take part in the „Coronataxi" study by the local health departments in the vicinity of Heidelberg. Therefore, this study is based on participants who volunteered. Inclusion criteria were age > 18, informed consent and registration in the Huma Medopad App for monitoring COVID-19 disease. The recruitment took place from September 7th 2020 to May 17th 2021.
Ethics oversight	The local Ethics committee of the University of Heidelberg, Germany had approved data collection of the COVID-19 cohort and analysis of the obtained samples (reference number: S-324/2020). The patient recruitment for the AHT control cohort took place under the ethics vote with the number 141/2018BO2, dated May 4, 2022. Since the data were kept in Würzburg, Germany, there is a second ethics vote with the number 52/18.

Note that full information on the approval of the study protocol must also be provided in the manuscript.

Field-specific reporting

Please select the one below that is the best fit for your research. If you are not sure, read the appropriate sections before making your selection.

☒ Life sciences ☐ Behavioural & social sciences ☐ Ecological, evolutionary & environmental sciences

For a reference copy of the document with all sections, see nature.com/documents/nr-reporting-summary-flat.pdf

Life sciences study design

All studies must disclose on these points even when the disclosure is negative.

Sample size	No sample size calculation was performed in this study. Samples from the COVID-19 cohort were collected as part of the aforementioned study at Heidelberg University Hospital, Germany. In some cases, multiple blood samples were collected per patient. An aliquot of these

	samples was transferred to the University Hospital of Tübingen, Germany, under appropriate conditions for NMR studies. In total, 509 samples from 329 COVID-19 patients were analyzed in our study.
Data exclusions	Quality control reports (B.I. BioBank QC) of the individual samples were checken. These quality control reports contain information about the quality of the NMR spectra, quality of the sample preparation and preanalytics, and possible contamination. A total of 26 samples of the COVID–19 cohort were sorted out based on these, mainly due to a linewidth of > 2.3 Hz, which indicates a poor quality of the NMR spectra. In the AHT control cohort, there were no data exclusions.
Replication	The NMR measurments and data analysis was performed by following the standard operation procedures (SOP), which guarantee a high reproducibility and without need of technical replicates.
Randomization	In this study, NMR data of various cohorts and subgroups of these cohorts were compared. The AHT control cohort was assembled age and sex matched to the COVID-19 cohort. For statistical analysis, the cohorts were classified according to criteria like sex, per-conditions, and disease progression. We didn't perform other experimental trails, therefore no further randomization was needed.
Blinding	This was a non–interventional study, therefore blinding was not relevant.

Reporting for specific materials, systems and methods

We require information from authors about some types of materials, experimental systems and methods used in many studies. Here, indicate whether each material, system or method listed is relevant to your study. If you are not sure if a list item applies to your research, read the appropriate section before selecting a response.

Materials & experimental systems

n/a	Involved in the study
☒	☐ Antibodies
☒	☐ Eukaryotic cell lines
☒	☐ Palaeontology and archaeology
☒	☐ Animals and other organisms
☒	☐ Clinical data
☒	☐ Dual use research of concern

Methods

n/a	Involved in the study
☒	☐ ChIP-seq
☒	☐ Flow cytometry
☒	☐ MRI-based neuroimaging
